# Identification and validation of predictive factors for progression to severe COVID-19 pneumonia by proteomics

**DOI:** 10.1038/s41392-020-00333-1

**Published:** 2020-10-03

**Authors:** Biao Di, Hongling Jia, Oscar Junhong Luo, Fangqin Lin, Kuibiao Li, Yuanliang Zhang, Huadong Wang, Huiying Liang, Jun Fan, Zhicong Yang

**Affiliations:** 1 Guangzhou Center for Disease Control and Prevention, 510440 Guangzhou, Guangdong China; 2 Department of Medical Biochemistry and Molecular Biology, School of Medicine, Jinan University, 510632 Guangzhou, Guangdong China; 3 Department of Systems Biomedical Sciences, School of Medicine, Jinan University, 510632 Guangzhou, Guangdong China; 4 GMU-GIBH Joint School of Life Sciences, Guangzhou Medical University, 511436 Guangzhou, Guangdong China; 5 BGI-Shenzhen. Beishan Industrial Zone, 11th building, Yantian District, 518055 Shenzhen, Guangdong China; 6 Department of Pathophysiology, Key Laboratory of National Administration of Traditional Chinese Medicine of the People’s Republic of China, School of Medicine, Jinan University, 510632 Guangzhou, Guangdong China; 7 Clinical Data Center, Guangdong General Hospital/Guangdong Academy of Medical Sciences, 510080 Guangzhou, Guangdong China

**Keywords:** Predictive markers, Molecular biology

**Dear Editor,**

Recent study showed that around 80% of coronavirus disease-19 (COVID-19) patients are moderate cases who will recover with or without conventional treatment, while the remaining 20% developed severe disease requiring intensive care.^1^ Early and accurate screening of new COVID-19 patients to identify those who will develop severe disease will facilitate decision-making on appropriate treatment regimens and reasonable allocation of limited healthcare resources. Therefore, novel predictive factors for disease progress from moderate to severe are urgently needed.

As proteomic profiling from sera of patients are informative during the disease progression, we hypothesize that some proteins would be significantly altered in the sera of patients who will develop severe COVID-19 after admission, which are informative to predict the disease progression.^2,3^ Herein, we initiated a study by recruiting two groups of COVID-19 patients, the development group (group I) with 23 patients (15 severe and 8 moderate) (Supplementary Table S1); and the validation group (group II) with 50 patients (29 severe and 21 moderate) (Supplementary Table S2) (Fig. S1a). In addition, 10 healthy individuals negative for the SARS-CoV-2 nucleic acid test were recruited as control group. To characterize the proteomics signatures of COVID-19 patients, we performed a quantitative liquid chromatography-tandem mass spectrometry/mass spectrometry (LC-MS/MS) proteomic analysis on the sera of the patients from group I and the control group. Overall, the abundance of 988 proteins across these 33 individuals exhibited high consistency (Supplementary Fig. 1b; Supplementary Data S1). Based on the treatment records, 15 patients in group I who required ventilator were classified as severe cases, and the rest were classified as moderate (Supplementary Table S1). Principal component analysis (PCA) results show that although most of the moderate patients are similar to healthy controls, the severe patients clearly segregated from the rest (Fig. 1a), suggesting some unique confounding proteomic features for the severe cases. Differential expression (DE) analysis between patients and healthy controls identified 43 and 47 significantly upregulated and downregulated proteins, respectively (Fig. 1b, c, Supplementary Table S3). The upregulated proteins include classical inflammatory response proteins, such as protein S100-A8 (S100A8), protein S100-A9 (S100A9), serum amyloid A-1 protein (SAA1), serum amyloid A-2 protein (SAA2), and alpha-1-antichymotrypsin (SERPINA3). We performed systematic Gene Ontology (GO) enrichment analysis and the results show that the upregulated proteins are significantly enriched (Bonferroni corrected *P* < 0.05) in biological processes related to acute inflammatory response, neutrophil activation, and activation of innate immune response (Fig. 1d; Supplementary Table S4). Our results also indicate that some of the upregulated proteins are significantly involved in response to hypoxia and cytokine secretion (Fig. 1d). The significance of some of these differentially expressed proteins were independently validated by parallel reaction monitoring (PRM) (Supplementary Figs. 2, 3). These results provide an initial proteomics characterization for the 23 confirmed COVID-19 patients. DE analysis between the severe and moderate cases show that 20 proteins are significantly upregulated in the severe cases (Fig. 1e, f, Supplementary Table S5). Ten of them, including S100A8 and S100A9, are also identified as upregulated in the previous comparison between the patients and the healthy counterparts. Upregulation of these 10 proteins identified in both comparisons suggests that the severe cases might have stronger inflammatory responses than the moderate patients. Upregulations of the other 10 proteins including actin, cytoplasmic 1 (ACTB), calpain small subunit 1 (CAPNS1), collagen alpha-3(VI) chain (COL6A3), coagulation factor VIII (F8), glutathione S-transferase omega-1 (GSTO1), myoglobin (MB), out at first protein homolog (OAF), superoxide dismutase [Mn], mitochondrial (SOD2), thymosin beta-4 (TMSB4X), and tumor necrosis factor receptor superfamily member 17 (TNFRSF17), are only found in severe patients while the expression of such those 10 proteins are either nonsignificantly different or downregulated in moderate patients as compared to controls (Supplementary Fig. 4). Functional enrichment analysis of the 20 upregulated proteins in the progression to severe group show that, in addition to immune response related biological processes, some of these proteins (ACTB, GSTO1, S100A9, OAF, and SOD2) are significantly involved in cellular detoxification during the disease progression. (Fig. 1g; Supplementary Table S6).

**Fig. 1:**
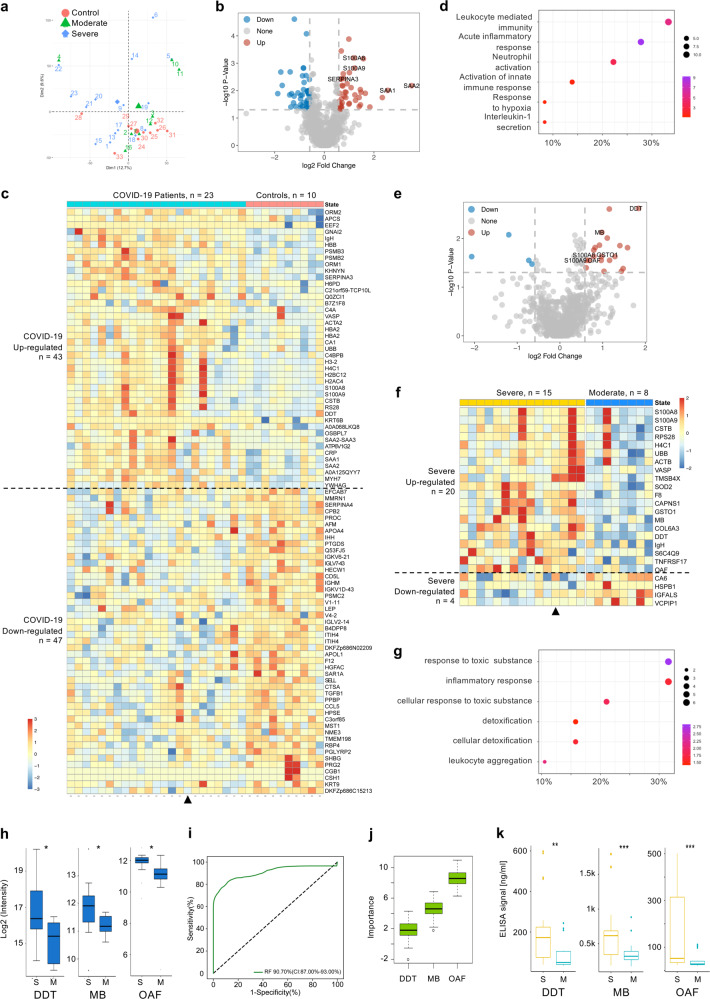
Identification of proteomic predictive factors for severe COVID-19 patient screening. Sera samples from two independent cohorts were collected. Samples from Development group were proteomically analyzed by LC-MS/MS and PRM. Machine-learning analysis of the proteomics data identified three predictive factors for screening severe COVID-19 patients. Samples from Validation group were analyzed by ELISA assay to validate the proteomic predictive factors. **a** PCA plot of the proteomics data (988 quantified proteins), red, green and blue data points are control, moderate and severe COVID-19 patients, respectively. The subject ID numbers are also shown. The centroid of each sample group is indicated by a larger solid red circle, green triangle and blue diamond, respectively. **b** Volcano plot showing DE protein identification between all patients (Development group) and controls. Selected DE proteins are indicated. **c** Heatmap visualization of the 90 significantly DE proteins between patients and controls. Upregulated and downregulated are in respect to patients vs. controls. Triangle indicates the only patient (Patient 22) who was classified as severe on hospital admission. Gene names are shown on the right. **d** Dotplot visualization of selected GO biological process terms for the upregulated proteins in COVID-19 patients. Dot sizes are proportional to the number of proteins annotated to the corresponding GO term. Color-scale is proportional to −log10(adjusted *P* value). **e** Volcano plot showing DE protein identification between all severe and moderate patients in Development group, with selected DE proteins indicated. **f** Heatmap visualization of the 24 significantly DE proteins between severe and moderate patients. Upregulated and downregulated are in respect to severe vs. moderate. Triangle indicates the only patient who was classified as severe on hospital admission. Gene names are shown on the right. **g** Dotplot visualization of selected GO biological process terms for the upregulated proteins in severe patients. Dot sizes are proportional to the number of proteins annotated to the corresponding GO term. Color-scale is proportional to −log10(adjusted *P* value). **h** Boxplots of PRM validation intensity of DDT, OAF, and MB in severe and moderate patients. **i** ROC of the random forest model built with the three protein features shown in **j**. The AUC is quoted with the 95% confidence interval values in brackets. **j** Importance ranking of OAF, MB and DDT in random forest models. **k** Boxplots of ELISA detection values of DDT, MB, and OAF between moderate (*n* = 21) and severe (*n* = 29) COVID-19 patients in Validation group. *P < 0.05. *P* values were calculated by Wilcoxon Rank Sum test. S Severe, M Moderate

To consolidate the specificity of the upregulated proteins in the severe patients, we performed additional PRM validation. The validation results indicate 8 (S100A8, OAF, 40S ribosomal protein S28 (RPS28), SOD2, MB, GSTO1, D-dopachrome decarboxylase (DDT) and CAPNS1) out of the 10 upregulated proteins are of significantly higher detection rate by PRM in the progression to severe samples than the moderate ones (*P* < 0.05, Wilcoxon rank sum test, Fig. 1h, Supplementary Figs. 5, 6, and 7). Next, we performed random forest machine-learning to test whether any of these factors with differential protein expression profiles can be used for early detection of potential severe COVID-19 patients. The results show that the combination of these eight proteins can only achieve an area-under-curve (AUC) of ~0.80 (Supplementary Fig. 8).

To further identify severe COVID-19 predictive factor proteins, we designed an iterative model building approach on the proteomics data to search for an optimal combination of proteins for prediction of severe COVID-19 patients (Supplementary Fig. 9a). All 255 combinations of the eight PRM validated protein features were exhaustively tested. The overall results indicate that the combination of OAF, MB, and DDT is the best predictor for screening severe patients with AUC = 0.907 (CI:0.87–0.93) (Fig. 1i, j, Supplementary Fig. 9b–d, Supplementary Table S7).

To validate the predictive performance of DDT, OAF, and MB, we further determined their protein levels in sera samples from patients in group II by enzyme-linked immunosorbent assay (ELISA) assay, which is more clinically and financially practical than LC-MS/MS and PRM methods, the results show that all of DDT, MB, and OAF exhibited significantly (*P* < 0.05) higher concentrations by ELISA in severe patients than moderate (Fig. 1k; Supplementary Fig. 10a). In addition, random forest analysis of the combined ELISA data of these three proteins also indicate a strong predictive power (AUC = 0.904, CI: 0.89–0.91) for severe patient detection (Supplementary Fig. 10b, c).

In addition to the potential to alert the disease progression, the identified predictive factors panel may also shed light on potential molecular mechanisms underlying COVID-19 progression. DDT, also recognized as MIF-2, may play a pivotal upstream role in the inflammatory cascade by promoting the secretion of other inflammatory mediators, including TNF-a, IL-6, which are considered as major cytokine involved in cytokine storm syndrome in COVID-19 patients.^4^ Rhabdomyolysis with significant increase of myoglobin was reported on COVID-19, suggesting that kidney injury/dysfunction is another fatal risk in severe patients with COVID-19.^5^ It would be more interesting to investigate the role of OAF (Out at first protein homolog) in the progression of COVID-19, which remains largely unknown. Generally, our study supports the concept hat severe COVID-19 is rather a systemic dysfunction than a sole severe viral pneumonia with respiratory failure.

In conclusion, we performed statistical analysis on the combined proteomic signatures of COVID-19 patients and successfully identified DDT, OAF, and MB as a panel of predictive factors to predict the potential deterioration of patients with moderate COVID-19 at admission before manifestation of severe symptoms. Our proposed predictive factors panel would be helpful for early identification of potential severe COVID-19 patients, which is not only critical to the lifesaving of patients with COVID-19 but also beneficial to relief of COVID-19 pandemic in most countries.

## Supplementary information

SUPPLEMENTAL MATERIAL

Data S1
