# Supplementary material for: Identification and validation of predictive factors for progression to severe COVID-19 pneumonia by proteomics
Source: Signal Transduct Target Ther. 2020 Oct 3;5:217. doi: 10.1038/s41392-020-00333-1 (PMC7532335; doi:10.1038/s41392-020-00333-1)
Supplement: Supplementary file 1 — SUPPLEMENTAL MATERIAL [file 41392_2020_333_MOESM1_ESM.pdf]

Supplementary Materials for

Identification and validation of predictive factors for progression to severe COVID-19 pneumonia by proteomics

Biao Di, Hongling Jia, Oscar Junhong Luo, Fangqin Lin, Kuibiao Li, Yuanliang Zhang, Huadong Wang, Huiying Liang, Jun Fan, Zhicong Yang

Correspondence to: Zhicong Yang (yangzc@gzcdc.org.cn) or Jun Fan (fanjun@jnu.edu.cn) or Huiying Liang (lianghuiying@hotmail.com)

This PDF file includes:

Materials and Methods
Figures. S1 to S10
Tables S1 to S7
Data S1. List of LC-MS/MS detected peptides and proteins from sera of 23 COVID-19 patients (Development group) and 10 healthy controls

Other Supplementary Materials for this manuscript include the following:

Data S1. List of LC-MS/MS detected peptides and proteins from sera of 23 COVID-19 patients (Development group) and 10 healthy controls

Materials and Methods

Study Oversight and Participants

Guangzhou Center for Disease Control and Prevention (Guangzhou CDC) has been authorized for laboratory testing for SARS-CoV-2 infections since the official announcement of COVID-19 breakout, according to Chinese Law for Infectious Disease and Law for Health and Quarantine. All the patients with laboratory confirmed SARS-CoV-2 infection were referred to the No. 8 People's Hospital of Guangzhou, which specializes in infectious diseases and has been designated as one of the major designated hospitals to treat COVID-19 patients in the province.

Between January 27 and March 3, 2020, we recruited 73 adult COVID-19 patients who were laboratory confirmed at Guangzhou CDC laboratory and then were referred to the No. 8 People's Hospital of Guangzhou for treatment. All patients were divided into development group (Group I: 23 patients recruited between January 27 and February 10) and validation group (Group II: 50 patients recruited between February 13 and March 3). A total of 10 healthy volunteers were recruited as control group. The diagnosis of COVID-19 was made following the Protocol for Novel Coronavirus Pneumonia Diagnosis and Treatment issued by the National Health Commission of the People's Republic of China. Written informed consent was obtained from each participant and the present study was approved by the Ethical Committee of Guangzhou CDC (GZCDC-ECHR-2020A0002).

Sample and Clinical Data collection

For all included COVID-19 patients (both Group I and Group II), serum samples and a panel of epidemiological, clinical, radiological and laboratory data were collected within 24 hours after admission. In addition, infection with other respiratory pathogens such as influenza A virus (H1N1, H3N2, H7N9), influenza B virus, respiratory syncytial virus (RSV), parainfluenza virus, adenovirus, SARS coronavirus and middle east respiratory syndrome (MERS) coronavirus were ruled out by real time - polymerase chain reaction (RT-PCR) assays approved by the China Food and Drug Administration. In addition, blood serum samples of group I and healthy control were collected for quantitative data-independent acquisition (DIA) proteomics analysis (Supplementary Fig. 1a). The serum samples were deactivated at 56 °C for 30 minutes before proteomics analysis.

Proteomics data analysis and confirmation

For deeper proteomic analysis, the proteins extracted from 100uL serum by 8 M urea (which can denature all proteins) and vortexed for 5 min. And then 10 mM dithiothreitol (DTT) was added to the mixture of serum and urea at 37℃ for 1 h followed by added 55 mM iodoacetamide (IAM) and incubated for 45 min in a dark room. Finally, the mixture was centrifuged at 25,000 × g for 20 min at 4℃, and the 1ml supernatant get through the Cleanert PEP96 wellplate inserted with 30mg C18 individual columns (Agela Technologies, China) that bind with the lower abundance serum proteins with higher hydrophobicity and expel the high-abundance proteins in the flowthrough. The bound lower abundance proteins were finally eluted with 75% ACN (Acetonitrile) (10.1021/acs.jproteome.9b00353). Sample was dehydrated in vacuum and re-dissolved in 50 mM Ammonium bicarbonate (ABC), digested with FASP (Sartorius, U.K.), at the ratio of 50:1 (protein to enzyme), subsequently eluded with 70% ACN and dehydrated in a vacuum centrifuge. DIA proteomic strategy was adopted to quantify the serum protein changes between the patients and health controls, initiating in a data dependent acquisition (DDA) spectral library established by the separation of peptide mixtures from the 23 development cohort samples and 10 control samples (10 μg/sample) into 10 fractions on an high performance liquid chromatography

(HPLC) system (Shimadzu, Japan) linked with Gemini high pH C18 column (4.6 × 250 mm, 5 μm) under a basic condition (pH 9.8). The peptides of each fraction pooled with iRT peptides were analyzed by Orbitrap Fusion Lumos Mass Spectrometer (Thermo Fisher Scientific, CA, USA) and identified by database searching software Maxquant (version 15.3.30) against human uniport protein database. The data acquisition of individual serum peptide sample pooled with the same amount of iRT peptides was performed under the DIA mode as reported and the data analysis was completed by Spectronaut (12.0.20491.14.21367) software referenced as the DDA library above. The proteins quantified with no less than 1.5-fold changes and P-value < 0.05 (Wilcoxon rank sum test) between case and control were defined as the differential proteins. To confirm the alterations of these differential proteins, a target quantification approach, PRM (Parallel Reaction Monitoring), was adopted for further analysis. For PRM confirmation, the 72 peptide precursors of selected differential proteins and iRT precursors were scheduled with 16-min retention time windows using Q-Exactive HF mass spectrometer (Thermo Fisher Scientific, CA, USA). The PRM quantification was performed by Skyline software and the intensities of three iRT peptides DGLDAASYYAPVR, GAGSSEPVTGLDAK, TPVISGGPYEYR were used to normalize the sample loading.

Validation of predictive factor performance

The accuracy of predictive factor panel was validated in the sera from group II and healthy controls by an ELISA assay. These ELISA kits are purchased from Beijing Andy Gene Co., Ltd and their commercial catalogue number are S100A8 (Cat: AD11500Hu), OAF (Cat: AD12688Hu), RPS28 (Cat: AD12692Hu), SOD2 (Cat: AD12691Hu), MB (Cat: AD12155Hu), GSTO1 (Cat: AD12018Hu), DDT (Cat: AD10014Hu) and CAPNS1 (Cat: AD12690Hu) respectively. The concentrations of these three proteins were determined according to the manufacturer's protocols. Absorbance was measured at 450 nm using HydroFlex microplate washer. All samples were analyzed in triplicate, and the average concentration for each patient was calculated.

Statistical analysis and machine-learning

Based on the disease progression, all 73 patients were classified as progression to severe group and moderate group. Categorical variables were summarized in percentage and compared between progression to severe group and moderate group using χ^2 test and Fisher's exact test. Continuous variables were expressed as median and interquartile range (IQR), whichever deemed appropriate. All data were analyzed with R (v3.5) and analysis of differentially expressed proteins was performed with MSstats R package which includes log2 transformation, normalization and *P*-value calculation on the Spectronaut and skyline quantitative data. PCA was performed with pcaMethods R package. GO enrichment analysis was performed with topGO R package with default parameters.

An iterative random forest machine-learning with 5-fold cross-validation approach was designed to identify the optimal panel of clinical and proteomic features for early detection of severe patient. Briefly, selected clinical and proteomic features from the patients were extracted for random forest modeling with 5-fold cross-validation, using disease severity as the response variable. Five-fold cross-validation was employed to eliminate potential overfitting of the random forest model. To reduce potential variability of the random forest models, each round of random sampling for training and testing dataset in model building was iteratively repeated for 100 times, and all reported results were averaged. All possible combinations of the selected clinical and proteomic features were exhaustively tested by this iterative machine-learning process, and the predictive power of each combination was recorded and ranked according to the AUC value by receiver operating characteristic (ROC) analysis. The average sensitivity and specificity scores

from each feature combination were also recorded. The combination of features with the highest AUC value was considered the best for distinguishing severe from moderate patients, and individual feature can be ranked according to the importance score in the corresponding random forest model.

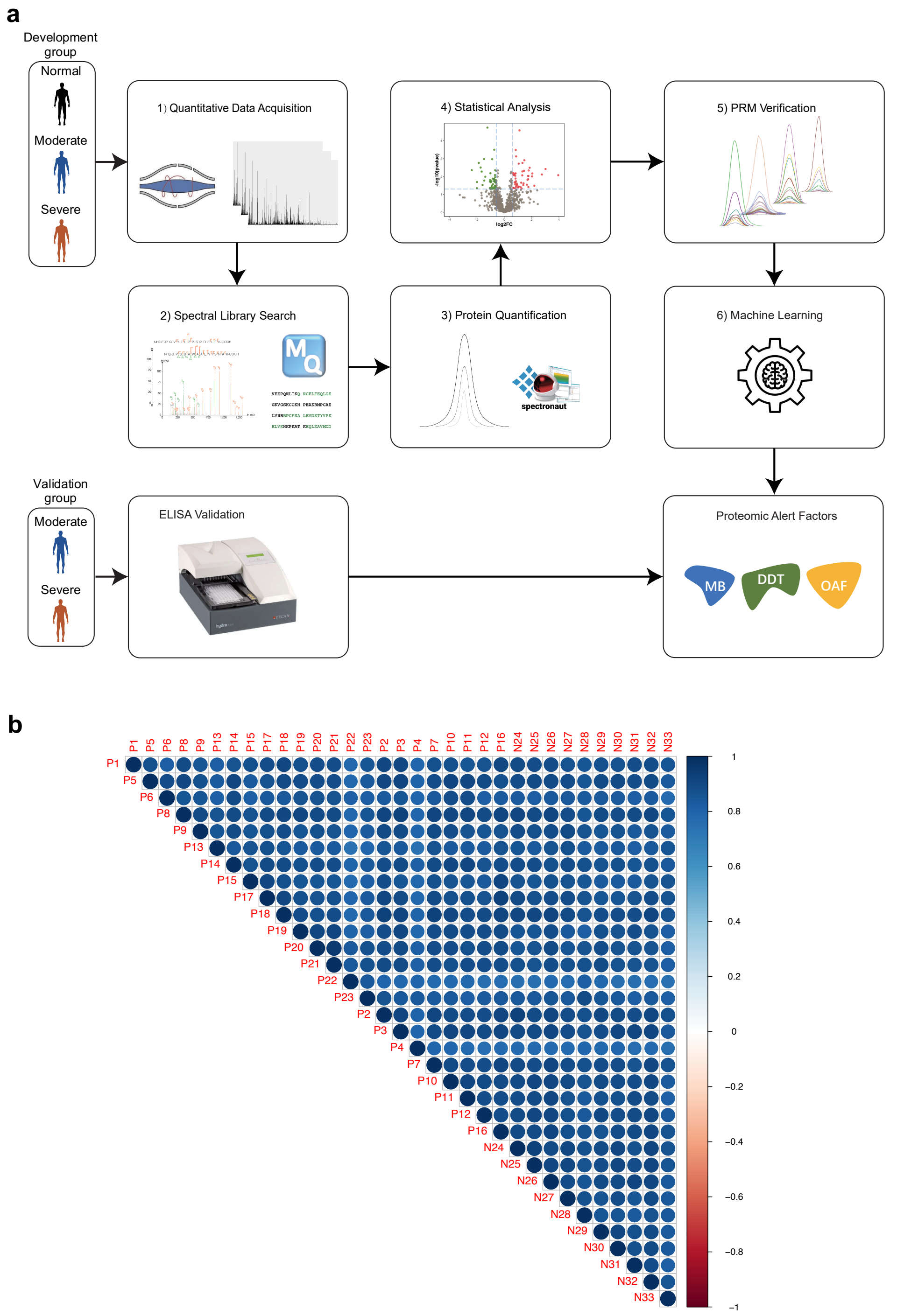

Figure. S1. Overall study design and data reproducibility assessment.

a Identification of proteomic predictive factors for severe COVID-19 patient screening. **b** Pearson's correlation of LC-MS/MS proteomics data among all 33 samples. P: Patient. N: Normal control.

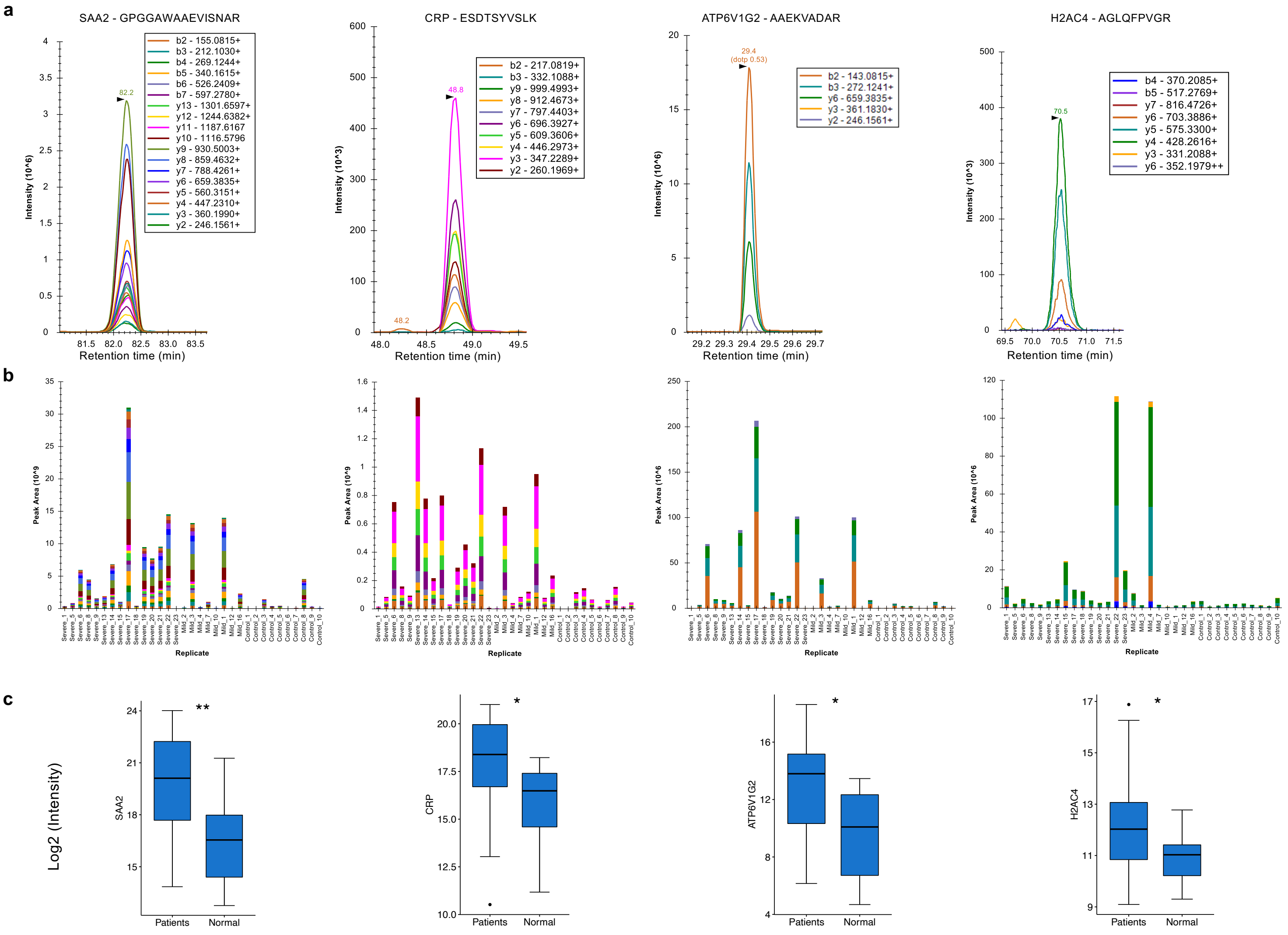

Figure. S2. PRM validation results for up-regulated proteins in COVID-19 patients.

a Validation of target peptides of SAA2, CRP, ATP6V1G2 and H2AC4 by PRM. **b** Cumulative bar plots of peak area of retention curves in a for patients and controls. Color code is same as a. **c** Boxplots of PRM detection intensity of SAA2, CRP, ATP6V1G2 and H2AC4 in patients and controls. * P-value < 0.05, ** P-value < 0.01, Wilcoxon Rank Sum test.

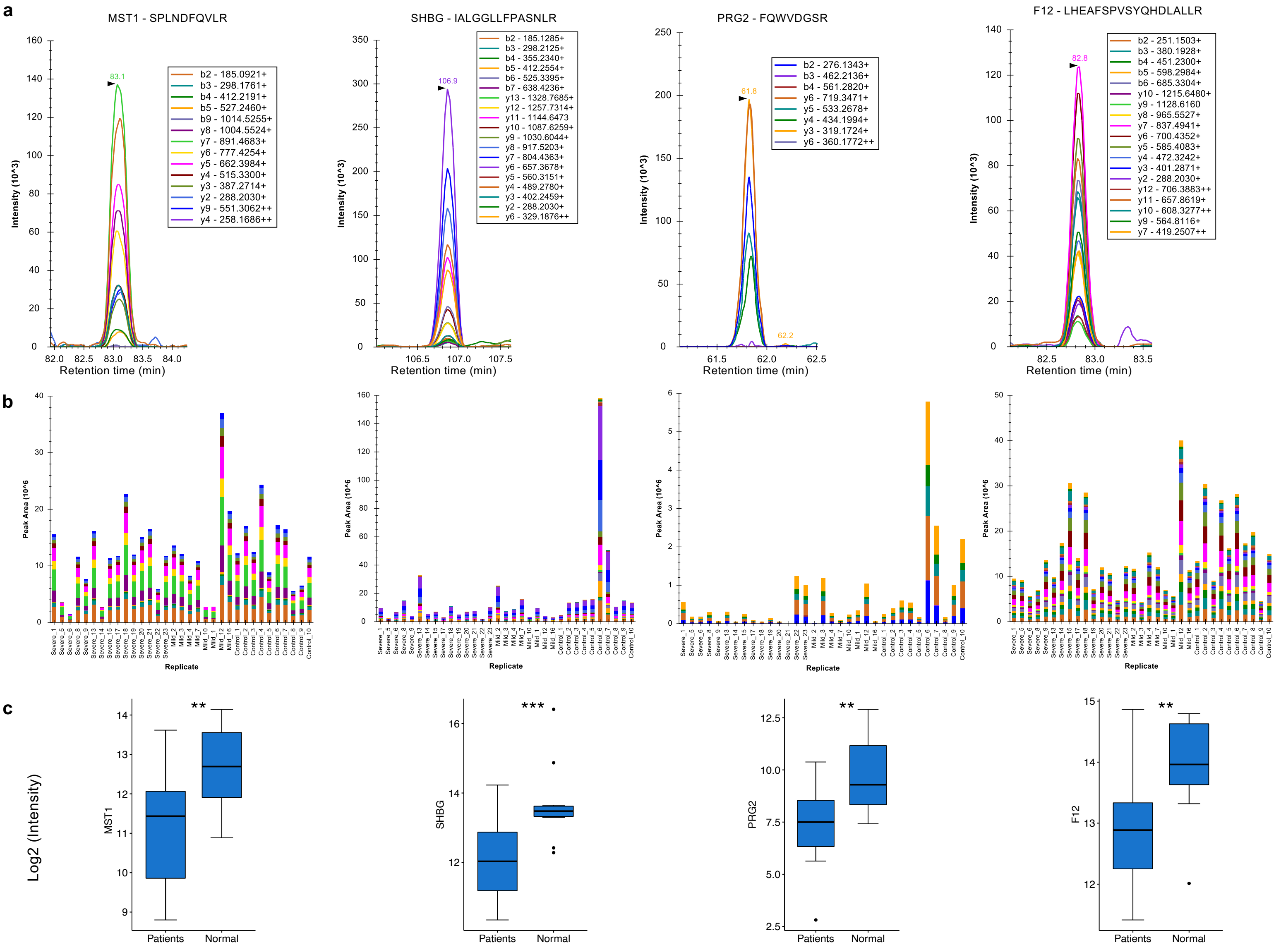

Figure. S3. PRM validation results for down-regulated proteins in COVID-19 patients.

a Validation of targeted peptide of MST1, SHBG, PRG2 and F12 by PRM. **b** Cumulative bar plots of peak area of retention curves in **a** for patients and controls. Color code is same as **a**. **c** Boxplots of PRM detection intensity of MST1, SHBG, PRG2 and F12 in patients and controls. ** P-value < 0.01, *** P-value < 0.001, Wilcoxon Rank Sum test.

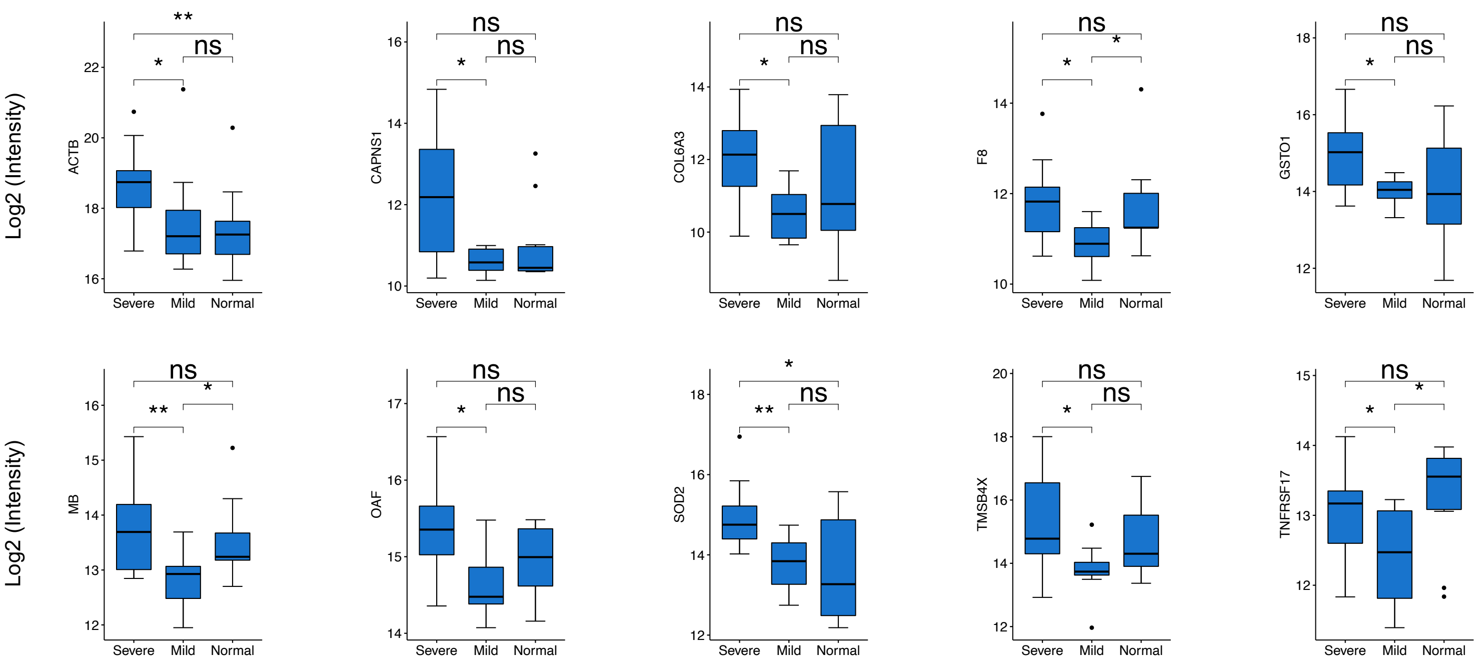

Figure. S4. Boxplots of PRM intensity for the 10 DE proteins between severe and moderate patients.

Data from the normal controls are also shown. All of these 10 proteins showed significant up-regulation in severe vs. moderate patient comparison. However, 7 of these proteins showed insignificant change between moderate patients and normal controls, and 3 even showed significant up-regulation in normal. * P-value < 0.05, ** P-value < 0.01, ns: not significant, Wilcoxon Rank Sum test.

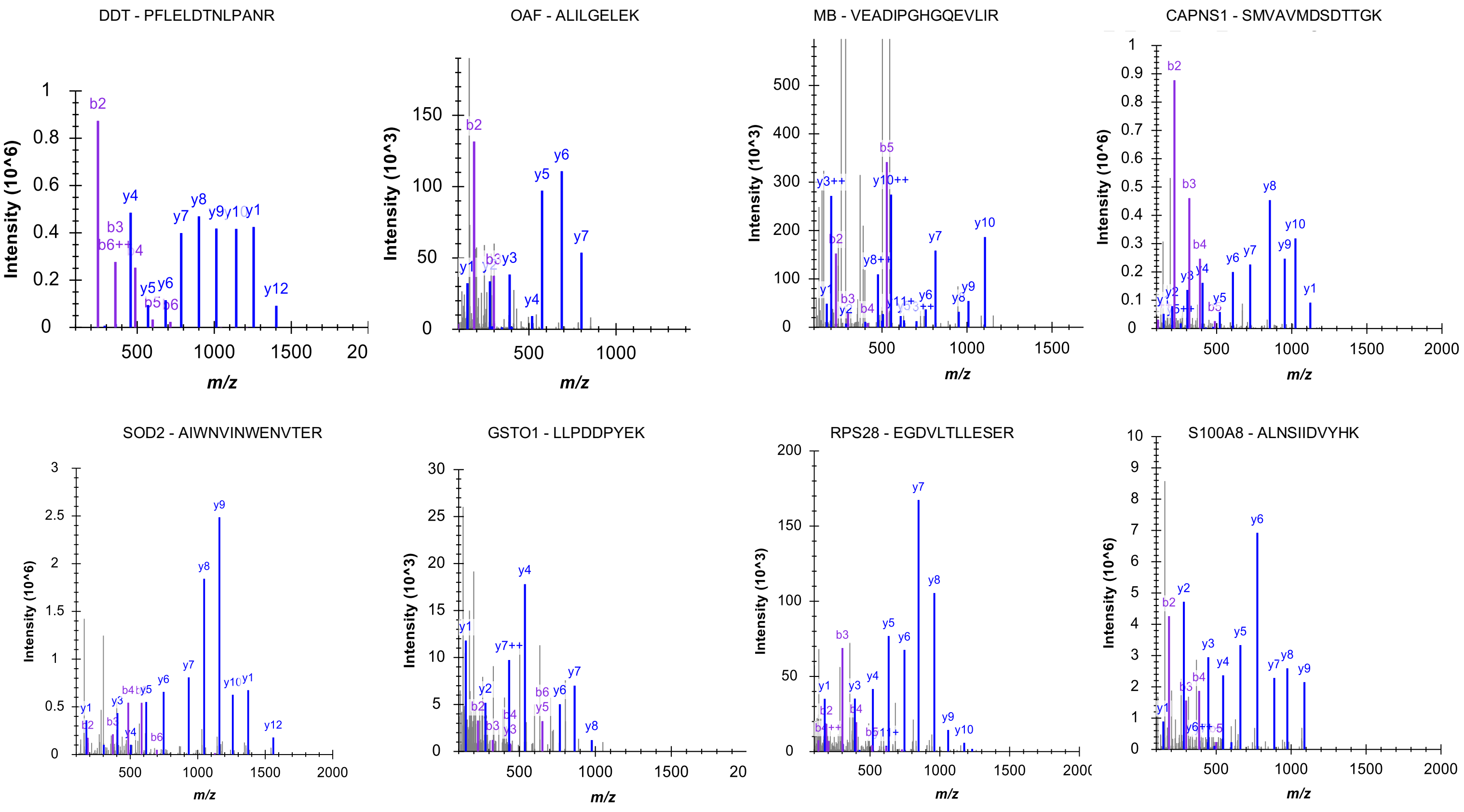

Figure. S5. MS/MS spectra for target peptides identified for DTT, OAF, MB, CAPNS1, SOD2, GSTO1, RPS28 and S100A8 by PRM.

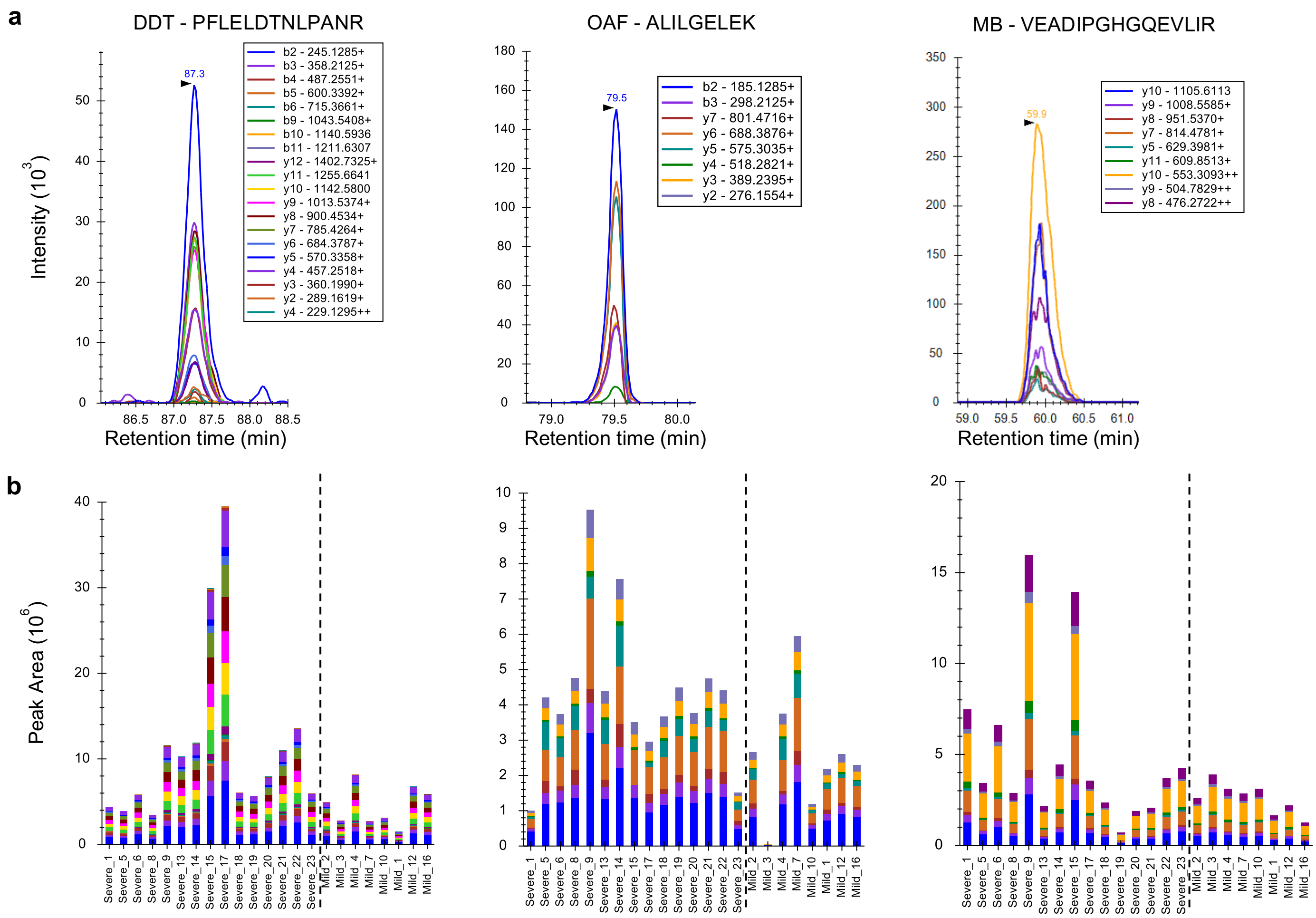

Figure. S6. PRM validation results for selected up-regulated proteins in severe COVID-19 patients.

a Validation of target peptides of DDT, OAF and MB by PRM. **b** Cumulative bar plots of peak area of retention curves in A for severe and moderate patients. Color code is same as **a**. Dashed vertical lines separates severe and moderate patients.

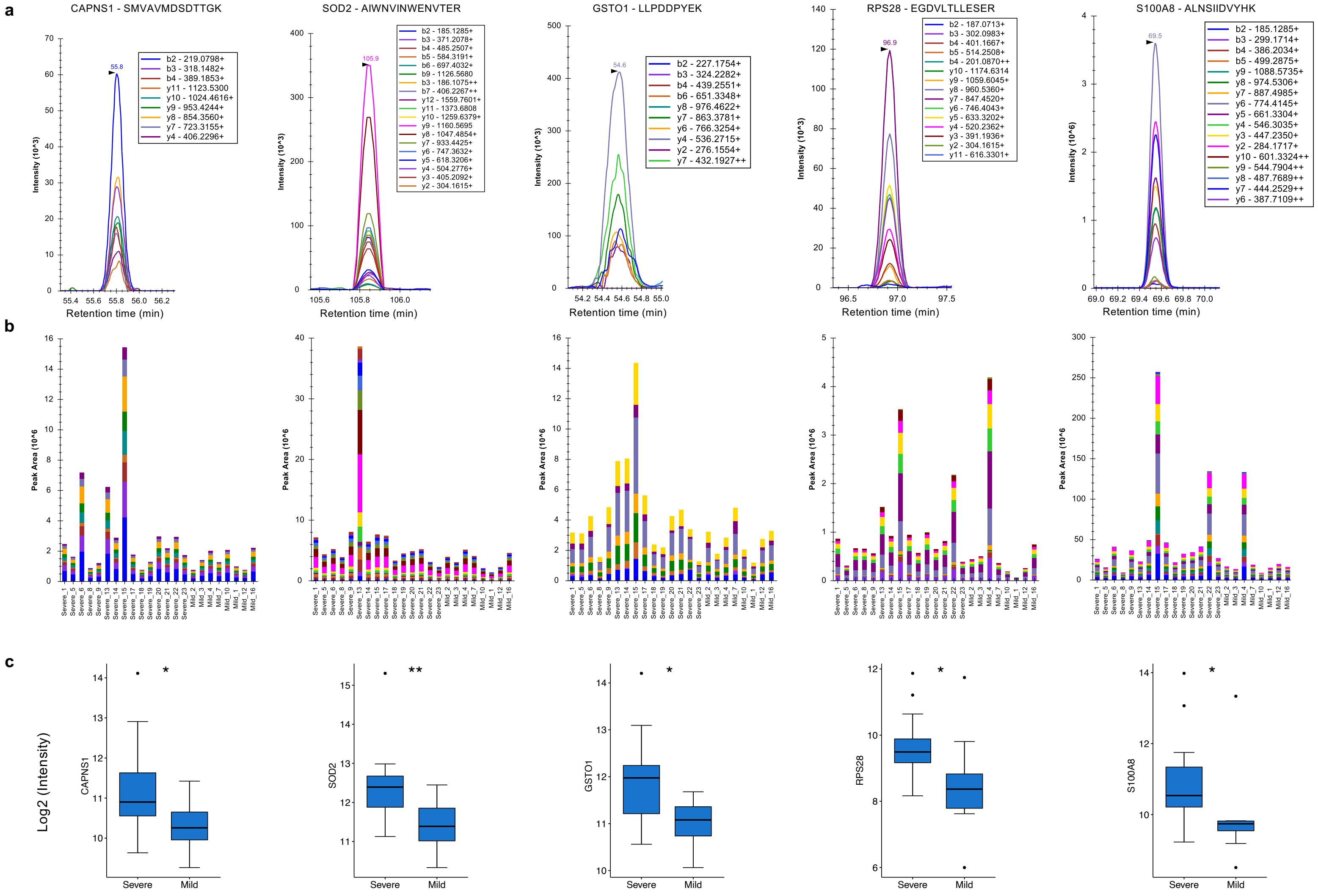

Figure. S7. PRM validation results for selected up-regulated proteins in severe COVID-19 patients.

a Validation of target peptides of CAPNS1, SOD2, GSTO1, RPS28 and S100A8 by PRM. **b** Cumulative bar plots of peak area of retention curves in a for severe and moderate patients. Color code is same as **a**. **c** Boxplots of PRM detection intensity of CAPNS1, SOD2, GSTO1, RPS28 and S100A8 in severe and moderate patients. * P-value < 0.05, ** P-value < 0.01, Wilcoxon Rank Sum test.

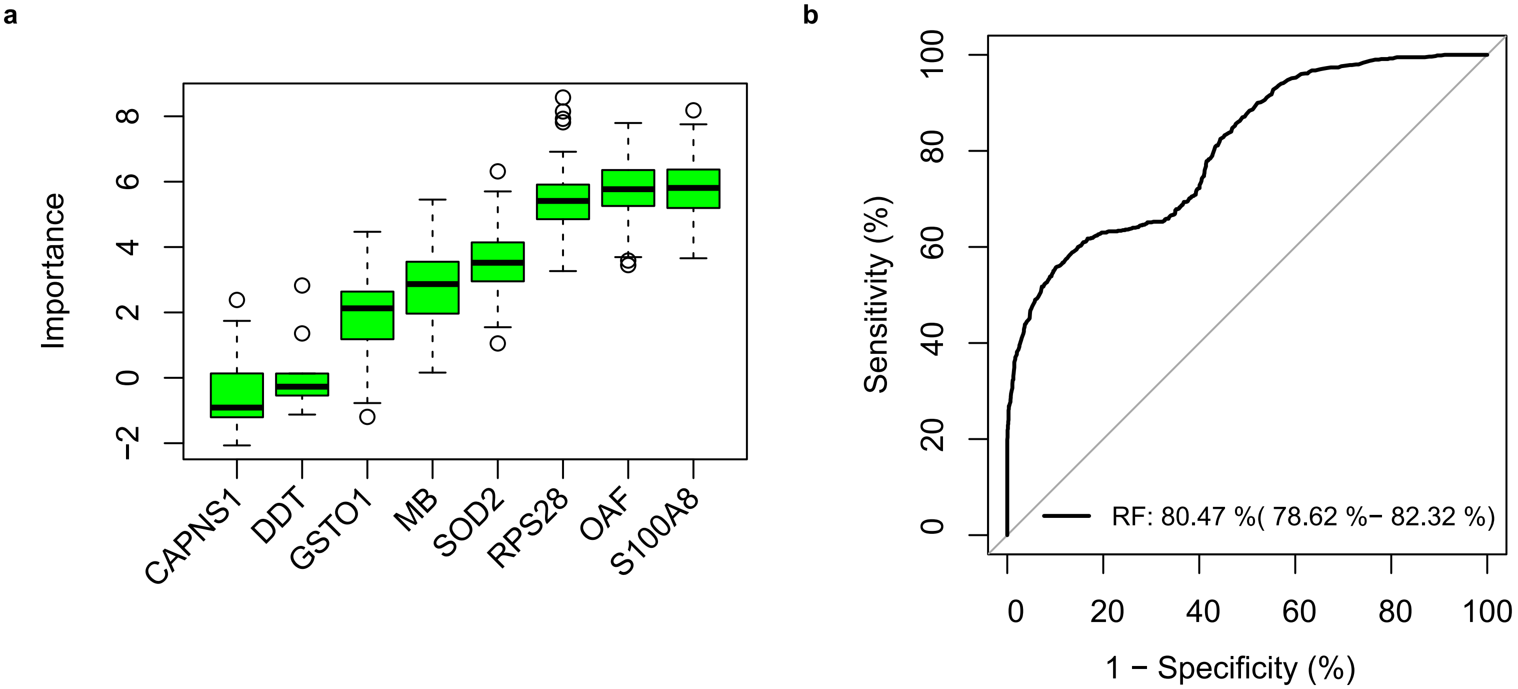

Figure. S8. Summary of random forest machine-learning results for selected DE proteins between severe and moderate COVID-19 patients.

a Importance ranking for the 8 selected severe patient specific proteins used for random forest model. **b** ROC of the random forest machine-learning model using proteomics data of the 8 selected proteins for screening severe patients among all patients. The AUC is quoted with the 95% confidence interval values in brackets.

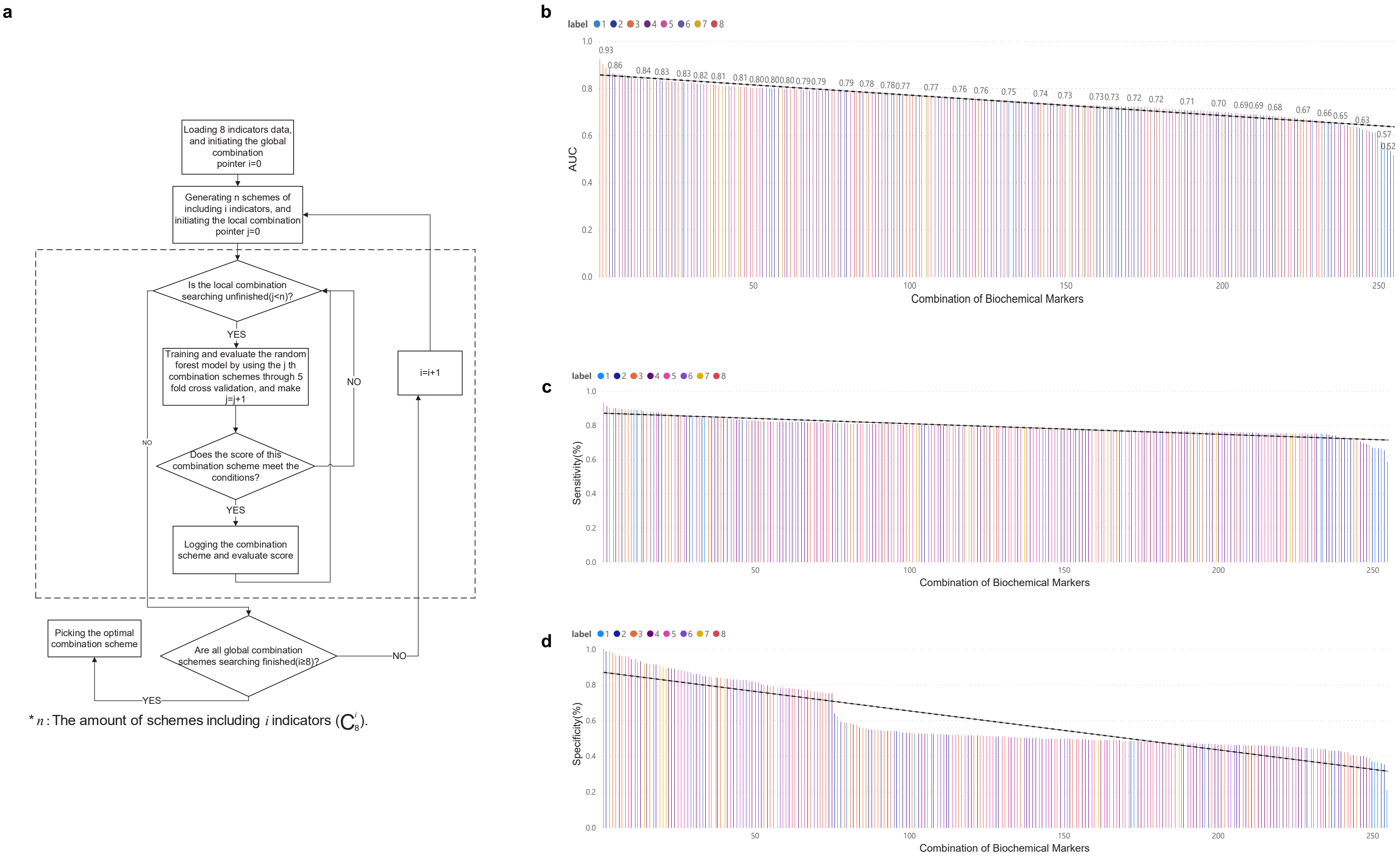

Figure. S9. Summary of the iterative random forest machine-learning leveraging proteomics data of distinct protein combinations.

a Workflow of the iterative random forest machine-learning approach. AUC (**b**), Sensitivity (**c**) and Specificity (**d**) ranking of all 255 random forest models with different number of features. The dashed lines indicate the fitted lines showing the AUC, sensitivity and specificity trend. The color-code indicates the number of features used for each random forest model building.

a

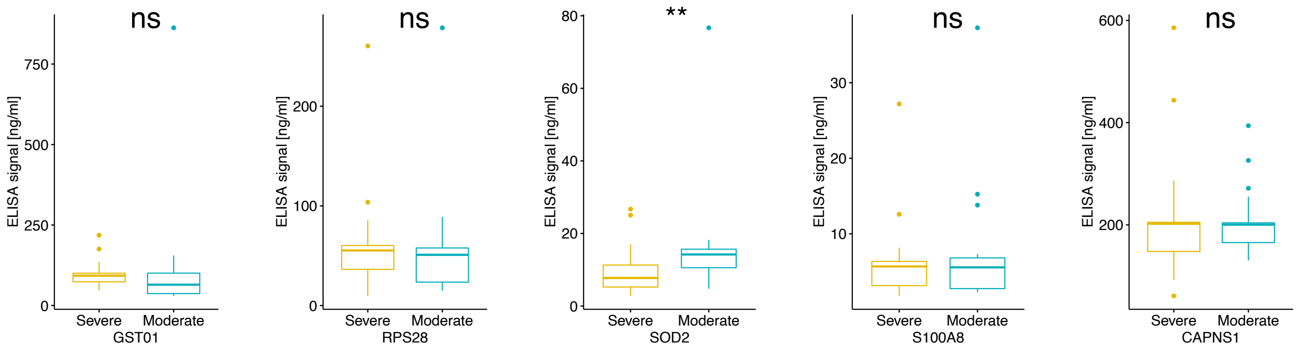

b

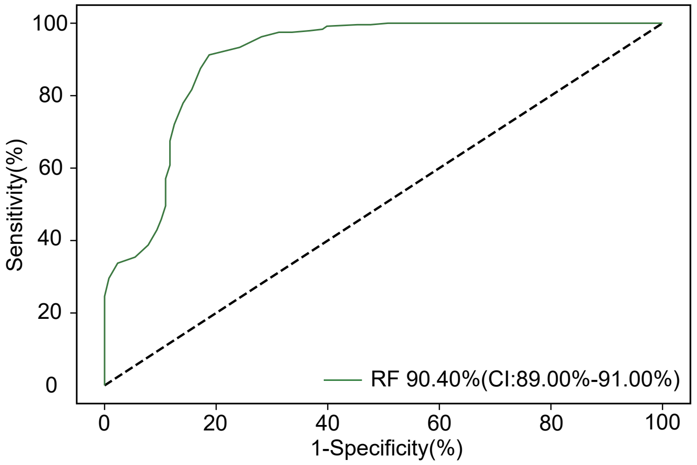

c

Confusion Matrix		Predict Labels: Stable Mild Cases	Predict Labels: Progression to Severe Cases
True Labels	Stable Mild Cases	18	3
True Labels	Progression to Severe Cases	4	25

Figure. S10. ELISA and Random forest machine-learning summary on ELISA validation results of DDT, MB and OAF with 50 COVID-19 patients in Validation group.

a Boxplots of ELISA detection values of GST01, RPS28, SOD2, S100A8 and CAPNS1 between moderate (n=21) and severe (n=29) COVID-19 patients. **b** ROC of the random forest model built with the ELISA data of DDT, MB and OAF. The AUC is quoted with the 95% confidence interval values in brackets. **c** Confusion matrix showing the classification result by random forest analysis with 5-fold cross-validation on the ELISA data. Majority of moderate (n=18) and severe (n=25) samples were correctly classified, indicating the ELISA results of DDT, MB and OAF were reliable in screening severe COVID-19 patients. ** P-value < 0.01, ns: not significant, Wilcoxon Rank Sum test.

Table S1. Clinical Characteristics at admission of 23 COVID-19 patients (Development group)

Characteristics	All Patients (n=23)	Moderate * (n=8)	Severe* (n=15)	*P*
Age, median (IQR), year	56.00(43.00-62.00)	46.00(43.00-58.00)	61.00(37.00-64.00)	0.39
Female sex	9(39.1%)	4(50.0%)	5(33.3%)	0.44
Coexisting disorder#	10(43.5%)	3(37.5%)	7(46.7%)	0.67
Clinical signs and symptoms				
Fever	18(78.3%)	6(75.0%)	12(80.0%)	1.00
Cough	18(78.3%)	7(87.5%)	11(73.3%)	0.62
Chilly	6(28.6%)	2(28.6%)	4(28.6%)	1.00
Myalgia	9(42.9%)	3(42.9%)	6(42.9%)	1.00
Fatigue	11(52.4%)	5(71.4%)	6(42.9%)	0.36
Rhinorrhoea	4(19.0%)	2(28.6%)	2(14.3%)	0.57
Shortness of breath	6(28.6%)	1(14.3%)	5(35.7%)	0.61
Sore throat	5(21.7%)	3(37.5%)	2(13.3%)	0.30
Nausea or vomiting	3(14.3%)	2(28.6%)	1(7.1%)	0.25
Diarrhea	2(8.7%)	0(0%)	2(13.3%)	0.53
Chest CT findings				
Ground-glass opacity	10(43.5%)	4(50.0%)	6(40.0%)	0.69
Local mottling shadow	4(17.4%)	1(12.5%)	3(20.0%)	1.00
Bilateral mottling shadow	11(47.8%)	4(50.0%)	7(46.7%)	1.00
Laboratory parameters				
Leucocytes($\times10^9$/L; NR:3.5-	4.48(3.93-6.31)	4.54(4.08-6.95)	4.29(3.90-6.31)	0.87
Lymphocytes($\times10^9$/L; NR:1.1-	1.07(0.86-1.56)	1.35(1.03-2.53)	0.99(0.62-1.53)	0.08
Neutrophils($\times10^9$/L; NR:1.8-	2.96(2.23-4.94)	2.66(2.27-4.57)	3.17(2.16-4.94)	0.39
Platelets($\times10^9$/L; NR:125.0-	193.00(145.00-	208.00(187.75-	173.00(134.00-208.00)	0.19
HGB(g/L; NR:130.0-175.0)	138.00(119.00-	141.00(124.50-	133.00(116.00-147.00)	0.36
APTT(s; NR:21.0-37.0)	40.20(36.10-42.40)	38.90(36.50-40.18)	40.70(36.10-43.10)	0.12
PT(s; NR:10.5-13.5)	13.70(13.30-14.50)	13.60(13.25-14.05)	13.80(13.30-14.70)	0.68
D-dimer(mg/L; NR:0-500)	1240.00(910.00-	950.00(625.00-	1560.00(1150.00-	0.03
Albumin (g/L; NR:40.0-55.0)	37.70(35.30-41.50)	40.95(36.50-43.65)	36.20(32.50-40.80)	0.13
ALT (U/L; NR:9.0-50.0)	26.30(18.30-41.00)	24.10(19.98-37.33)	33.30(18.20-44.90)	0.83
AST (U/L; NR:15.0-40.0)	22.40(17.40-33.70)	19.15(16.10-20.65)	25.30(19.00-39.40)	0.06
TB (μmol/L; NR:0.0-21.0)	11.02(8.76-17.50)	13.73(10.62-20.77)	10.73(7.31-17.50)	0.43
BUN (mmol/L; NR:3.6-9.5)	4.21(3.18-5.22)	4.33(3.31-4.86)	3.54(3.18-5.78)	0.94
Scr (μmol/L; NR:57.0-111.0)	64.70(51.40-72.00)	65.50(60.20-73.83)	63.30(48.60-72.00)	0.51
CK (U/L; NR:50.0-310.0)	58.00(44.00-100.50)	51.00(43.00-84.00)	61.50(44.00-151.75)	0.32
LDH (U/L; NR:120.0-250.0)	208.00(155.50-	158.00(139.00-	223.00(192.50-407.50)	0.06
CRP (mg/L; NR:0.0-5.0)	4.90(4.90-32.46)	4.90(4.90-21.39)	4.90(4.90-43.96)	0.59

*Classification of COVID-19 severity is based on whether the patient required intensive care during through treatments.
Data are presented in median (IQR), or n (%);
P values are derived from Rank sum test, χ^2 test, or Fisher's exact test;
#including Hypertension, Diabetes, and Hyperlipemia;
Abbreviations: NR=Normal Range, HGB=Haemoglobin, APTT=activated partial thromboplastin time, PT=prothrombin time, ALT=alanine aminotransferase, AST=aspartate aminotransferase, TB=Total bilirubin, BUN=blood urea nitrogen, Scr=serum creatinine, CK=creatine kinase, LDH=lact ate dehydrogenase, CRP= C reactive protein.

Table S2. Clinical Characteristics at admission of 50 COVID-19 patients (Validation group)

Characteristics	All Patients (n=50)	Moderate (n=21)	Severe (n=29)	*P*
Age, median (IQR), year	56.00(44.00-62.00)	47.00(44.00-59.00)	29.00(37.00-63.50)	0.38
Female sex	31(62.00%)	11(52.38%)	20(68.97%)	0.23
Coexisting disorder*	26(52.00%)	9(42.86%)	17(58.62%)	0.27
Clinical signs and symptoms				
Fever	39(78.00%)	15(71.43%)	24(82.76%)	0.54
Cough	40(80.00%)	19(90.48%)	21(72.41%)	0.22
Chilly	11(22.00%)	3(14.29%)	8(27.59%)	0.52
Myalgia	19(38.00%)	7(33.33%)	12(41.38%)	0.71
Fatigue	25(50.00%)	14(66.67%)	11(37.93%)	0.01
Rhinorrhoea	9(18.00%)	6(28.57%)	3(10.34%)	0.14
Shortness of breath	6(12.00%)	0	6(20.69%)	0.03
Sore throat	12(24.00%)	9(42.86%)	3(10.34%)	0.00
Nausea or vomiting	8(16.00%)	6(28.57%)	2(6.90%)	0.02
Diarrhea	4(8.00%)	0	4(13.79%)	0.07
Chest CT findings				
Ground-glass opacity	23(46.00%)	12(57.10%)	11(37.90%)	0.25
Local mottling shadow	7(14.00%)	2(9.50%)	5(17.20%)	0.68
Bilateral mottling shadow	24(48.00%)	10(47.60%)	14(48.30%)	1.00
Laboratory parameters				
Leucocytes($\times 10^9$/L; NR:3.5-	4.54(4.02-6.31)	4.60(4.05-6.52)	4.29(3.92-6.31)	0.82
Lymphocytes($\times 10^9$/L;	1.07(0.88-1.61)	1.32(1.02-2.79)	0.99(0.62-1.53)	0.00
Neutrophils($\times 10^9$/L; NR:1.8-	2.93(2.23-4.94)	2.83(2.23-4.01)	3.17(2.16-4.94)	0.09
Platelets($\times 10^9$/L; NR:125.0-	193.00(145.00-	220.00(186.00-	173.00(134.00-	0.04
HGB(g/L; NR:130.0-175.0)	138.00(119.75-	140.00(120.00-	133.00(116.50-	0.16
APTT(s; NR:21.0-37.0)	40.10(36.25-41.43)	38.90(37.30-40.10)	40.70(36.20-43.10)	0.10
PT(s; NR:10.5-13.5)	13.65(13.28-14.30)	13.50(13.20-13.90)	13.80(13.30-14.70)	0.36
D-dimer(mg/L; NR:0-500)	1195.00(910.00-	990.00(700.00-	1560.00(1196.00-	0.05
Albumin (g/L; NR:40.0-	37.90(35.30-41.63)	41.00(35.30-43.10)	36.20(32.50-40.80)	0.02
ALT (U/L; NR:9.0-50.0)	26.30(19.28-41.00)	25.40(21.43-41.00)	33.30(18.25-44.90)	0.22
AST (U/L; NR:15.0-40.0)	21.60(18.60-33.40)	19.20(16.70-20.80)	25.30(20.70-39.40)	0.00
TB (μmol/L; NR:0.0-21.0)	11.36(8.99-18.47)	14.59(10.75-22.45)	10.73(8.04-17.50)	0.41
BUN (mmol/L; NR:3.6-9.5)	4.33(3.19-5.32)	4.35(4.09-4.86)	3.54(3.18-5.78)	0.22
Scr (μmol/L; NR:57.0-111.0)	64.90(55.30-72.33)	66.10(58.70-76.30)	63.30(48.60-72.00)	0.47
CK (U/L; NR:50.0-310.0)	57.00(44.00-97.00)	51.00(43.00-84.00)	63.00(44.00-139.00)	0.00
LDH (U/L; NR:120.0-250.0)	208.00(158.00-	173.50(149.50-	225.00(198.00-	0.00
CRP (mg/L; NR:0.0-5.0)	4.90(4.90-32.46)	4.90(4.90-20.73)	4.90(4.90-43.96)	0.15

Data are presented in median (IQR), or n (%);

P values are derived from *t* test, χ^2 test, or *Fisher's* exact test;

*including Hypertension, Diabetes, and Hyperlipemia;

Abbreviations: NR=normal range, HGB=haemoglobin, APTT=activated partial thromboplastin time, PT=prothrombin time, ALT=alanine aminotransferase, AST=aspartate aminotransferase, TB=total bilirubin, BUN=blood urea nitrogen, Scr=serum creatinine, CK=creatine kinase, LDH=lact ate dehydrogenase, CRP= C reactive protein.

Table S3. 90 differentially expressed proteins between COVID-19 patient group I and controls.

Gene name	Protein Accession	Ratio(Patient/Control)	Up Or Down	Adjusted P-value	Protein Description
SAA2	P0DJI9	11.448	UP	0.0066	Serum amyloid A-2 protein
SAA1	P0DJI8	5.874	UP	0.0098	Serum amyloid A-1 protein
CRP	P02741	5.059	UP	0.0179	C-reactive protein
ATP6V1G2	O95670	3.573	UP	0.0397	V-type proton ATPase subunit G 2
H2AC4	P04908	3.147	UP	0.0125	Histone H2A type 1-B/E
HBB	Q9GZL9	2.896	UP	0.0012	Beta-globin (Fragment)
KRT6B	P04259	2.747	UP	0.0331	Keratin, type II cytoskeletal 6B
PSMB2	P49721	2.735	UP	0.0092	Proteasome subunit beta type-2
H3-2	Q5TEC6	2.690	UP	0.0143	Histone H3
H4C1	P62805	2.651	UP	0.0382	Histone H4
H2BC12	O60814	2.645	UP	0.0178	Histone H2B type 1-K
MYH7	P12883	2.393	UP	0.0227	Myosin-7
DDT	P30046	2.352	UP	0.0143	D-dopachrome decarboxylase
S100A9	P06702	2.340	UP	0.0015	Protein S100-A9
S100A8	P05109	2.290	UP	0.0006	Protein S100-A8
ACTA2	P62736	2.115	UP	0.0078	Actin, aortic smooth muscle
YWHAG	P61981	2.020	UP	0.0020	14-3-3 protein gamma
OSBPL7	Q9BZF2	2.011	UP	0.0293	Oxysterol-binding protein-related protein 7
A0A068LKQ8	A0A068LKQ8	1.988	UP	0.0310	Ig heavy chain variable region (Fragment)
ORM1	P02763	1.985	UP	0.0001	Alpha-1-acid glycoprotein 1
C21orf59-TCP10L	F8VZ95	1.893	UP	0.0056	Uncharacterized protein (Fragment)
RS28	P62857	1.835	UP	0.0115	40S ribosomal protein S28
UBB	P0CG47	1.823	UP	0.0179	Polyubiquitin-B
HBA2	G3V1N2	1.810	UP	0.0114	HCG1745306, isoform CRA_a
APCS	P02743	1.744	UP	0.0159	Serum amyloid P-component
PSMB3	P49720	1.728	UP	0.0036	Proteasome subunit beta type-3
ORM2	P19652	1.698	UP	0.0024	Alpha-1-acid glycoprotein 2
IgH	A0A2U8J905	1.675	UP	0.0068	Ig heavy chain variable region (Fragment)
CA1	P00915	1.650	UP	0.0279	Carbonic anhydrase 1
VASP	P50552	1.631	UP	0.0065	Vasodilator-stimulated phosphoprotein
Q0ZCI1	Q0ZCI1	1.626	UP	0.0404	Immunglobulin heavy chain variable region (Fragment)
HBA2	A0A1S5UZ39	1.615	UP	0.0201	Hemoglobin subunit alpha
GNAI2	P04899	1.606	UP	0.0004	Guanine nucleotide-binding protein G(i) subunit alpha-2
SAA2-SAA3	A0A0D6A0A8	1.598	Up	0.0423	Serum amyloid A protein
CSTB	P04080	1.595	UP	0.0328	Cystatin-B
KHNYN	O15037	1.592	UP	0.0038	Protein KHNYN
A0A125QYY7	A0A125QYY7	1.574	UP	0.0382	IBM-B1 heavy chain variable region (Fragment)
SERPINA3	P01011	1.573	UP	0.0038	Alpha-1-antichymotrypsin
B7Z1F8	B7Z1F8	1.566	UP	0.0477	cDNA FLJ53025, highly similar to Complement C4-B
EEF2	P13639	1.552	UP	0.0331	Elongation factor 2
H6PD	O95479	1.547	UP	0.0311	GDH/6PGL endoplasmic bifunctional protein
C4BPB	P20851	1.533	UP	0.0125	C4b-binding protein beta chain
C4A	P0C0L4	1.509	UP	0.0251	Complement C4-A
CPB2	Q96IY4	0.666	DOWN	0.0201	Carboxypeptidase B2
AFM	P43652	0.664	DOWN	0.0201	Afamin
EFCAB7	A8K855	0.663	DOWN	0.0466	EF-hand calcium-binding domain-containing protein 7
TMEM198	Q66K66	0.662	DOWN	0.0311	Transmembrane protein 198
C3orf85	A0A1B0GTC6	0.660	DOWN	0.0039	Uncharacterized protein C3orf85
PPBP	P02775	0.657	DOWN	0.0125	Platelet basic protein
CD5L	O43866	0.654	DOWN	0.0098	CD5 antigen-like
CSH1	P0DML2	0.649	DOWN	0.0351	Chorionic somatomammotropin hormone 1
SELL	P14151	0.649	DOWN	0.0143	L-selectin
NME3	Q13232	0.648	DOWN	0.0125	Nucleoside diphosphate kinase 3
V4-2	Q5NV82	0.641	DOWN	0.0125	V4-2 protein (Fragment)

MMRN1	Q13201	0.633	DOWN	0.0311	Multimerin-1
PROC	P04070	0.630	DOWN	0.0103	Vitamin K-dependent protein C
APOL1	O14791	0.623	DOWN	0.0251	Apolipoprotein L1
DKFZp686C15213	Q6MZU6	0.622	DOWN	0.0102	Uncharacterized protein DKFZp686C15213
IGLV7-43	P04211	0.622	DOWN	0.0225	Immunoglobulin lambda variable 7-43
TGFB1	P01137	0.621	DOWN	0.0111	Transforming growth factor beta-1 proprotein
PSMC2	P35998	0.611	DOWN	0.0293	26S proteasome regulatory subunit 7
APOA4	P06727	0.608	DOWN	0.0423	Apolipoprotein A-IV
IGHM	P01871	0.608	DOWN	0.0001	Immunoglobulin heavy constant mu
KRT9	P35527	0.603	DOWN	0.0423	Keratin, type I cytoskeletal 9
PGLYRP2	Q96PD5	0.601	DOWN	0.0028	N-acetylmuramoyl-L-alanine amidase
IHH	Q14623	0.589	DOWN	0.0466	Indian hedgehog protein
SERPINA4	P29622	0.569	DOWN	0.0050	Kallistatin
CTSA	P10619	0.561	DOWN	0.0092	Lysosomal protective protein
Q53FJ5	Q53FJ5	0.560	DOWN	0.0015	Prosaposin (Variant Gaucher disease and variant metachromatic leukodystrophy) variant (Fragment)
V1-11	Q5NV67	0.559	DOWN	0.0279	V1-11 protein (Fragment)
IGKV6-21	A0A0C4DH24	0.559	DOWN	0.0015	Immunoglobulin kappa variable 6-21
IGKV1D-43	A0A0B4J1Z2	0.545	DOWN	0.0029	Immunoglobulin kappa variable 1D-43
IGLV2-14	A0A449C188	0.542	DOWN	0.0143	IGLV2-14*01_S1338 (Fragment)
CGB1	A6NKQ9	0.542	DOWN	0.0374	Choriogonadotropin subunit beta variant 1
SAR1A	Q9NR31	0.542	DOWN	0.0360	GTP-binding protein SAR1a
LEP	P41159	0.516	DOWN	0.0327	Leptin
CCL5	P13501	0.516	DOWN	0.0201	C-C motif chemokine 5
RBP4	P02753	0.515	DOWN	0.0423	Retinol-binding protein 4
HPSE	Q9Y251	0.472	DOWN	0.0086	Heparanase
HECW1	Q76N89	0.449	DOWN	0.0378	E3 ubiquitin-protein ligase HECW1
PTGDS	P41222	0.434	DOWN	0.0000	Prostaglandin-H2 D-isomerase
ITIH4	Q14624	0.431	DOWN	0.0159	Inter-alpha-trypsin inhibitor heavy chain H4
B4DPP8	B4DPP8	0.424	DOWN	0.0279	cDNA FLJ53075, highly similar to Kininogen-1
HGFAC	Q04756	0.401	DOWN	0.0129	Hepatocyte growth factor activator
DKFZp686N02209	Q7Z351	0.393	DOWN	0.0397	Uncharacterized protein DKFZp686N02209
F12	P00748	0.386	DOWN	0.0021	Coagulation factor XII
ITIH4	B7ZKJ8	0.381	DOWN	0.0143	ITIH4 protein
PRG2	P13727	0.309	DOWN	0.0065	Bone marrow proteoglycan
SHBG	P04278	0.297	DOWN	0.0021	Sex hormone-binding globulin
MST1	P26927	0.273	DOWN	0.0129	Hepatocyte growth factor-like protein

Table S4. Significantly enriched GO biological process terms for COVID-19 patient-specific proteins.

GO.ID	Term	Annotated	Significant	Expected	P value	Adjusted P	Significant genes
GO:0006953	acute-phase response	47	7	0.09	3.90E-12	3.90E-10	APCS,CRP,ORM1,ORM2,SAA1,SAA2,SERPINA3
GO:0002526	acute inflammatory response	218	10	0.43	9.80E-12	9.80E-10	APCS,C4A,C4BPB,CRP,ORM1,ORM2,S100A8,SAA1,SAA2,SERPINA3
GO:0006954	inflammatory response	875	12	1.74	6.20E-08	6.20E-06	APCS,C4A,C4BPB,CRP,DDT,ORM1,ORM2,S100A8,S100A9,SAA1,SAA2,SERPINA3
GO:0002443	leukocyte mediated immunity	878	12	1.75	6.40E-08	6.40E-06	APCS,C4A,C4BPB,CRP,CSTB,EEF2,HBB,ORM1,ORM2,S100A8,S100A9,SERPINA3
GO:0006952	defense response	1835	16	3.65	1.10E-07	1.10E-05	APCS,C4A,C4BPB,CRP,DDT,H2BC12,ORM1,ORM2,PSMB2,PSMB3,S100A8,S100A9,SAA1,SAA2,SERPINA3,UBB
GO:0002376	immune system process	3229	20	6.43	3.40E-07	3.40E-05	APCS,C4A,C4BPB,CRP,CSTB,DDT,EEF2,H2BC12,H4-16,H4C1,HBB,ORM1,ORM2,PSMB2,PSMB3,S100A8,S100A9,SAA1,SERPINA3,UBB
GO:0006955	immune response	2292	17	4.56	3.80E-07	3.80E-05	APCS,C4A,C4BPB,CRP,CSTB,EEF2,H2BC12,HBB,ORM1,ORM2,PSMB2,PSMB3,S100A8,S100A9,SAA1,SERPINA3,UBB
GO:0006950	response to stress	4178	22	8.32	9.50E-07	9.50E-05	APCS,C4A,C4BPB,CRP,DDT,EEF2,H2BC12,H4-16,H4C1,HBA2,HBB,MYH7,ORM1,ORM2,PSMB2,PSMB3,S100A8,S100A9,SAA1,SAA2,SERPINA3,UBB
GO:0002682	regulation of immune system process	1686	14	3.36	1.80E-06	0.00018	APCS,C4A,C4BPB,CRP,DDT,H4-16,H4C1,ORM1,ORM2,PSMB2,PSMB3,S100A8,S100A9,UBB
GO:0045087	innate immune response	1015	11	2.02	2.60E-06	0.00026	APCS,C4A,C4BPB,CRP,H2BC12,PSMB2,PSMB3,S100A8,S100A9,SAA1,UBB
GO:0002252	immune effector process	1276	12	2.54	3.50E-06	0.00035	APCS,C4A,C4BPB,CRP,CSTB,EEF2,HBB,ORM1,ORM2,S100A8,S100A9,SERPINA3
GO:0043312	neutrophil degranulation	483	8	0.96	3.70E-06	0.00037	CSTB,EEF2,HBB,ORM1,ORM2,S100A8,S100A9,SERPINA3
GO:0070488	neutrophil aggregation	2	2	0	3.80E-06	0.00038	S100A8,S100A9
GO:0002283	neutrophil activation involved in immune response	486	8	0.97	3.90E-06	0.00039	CSTB,EEF2,HBB,ORM1,ORM2,S100A8,S100A9,SERPINA3
GO:0002446	neutrophil mediated immunity	496	8	0.99	4.60E-06	0.00046	CSTB,EEF2,HBB,ORM1,ORM2,S100A8,S100A9,SERPINA3
GO:0042119	neutrophil activation	497	8	0.99	4.60E-06	0.00046	CSTB,EEF2,HBB,ORM1,ORM2,S100A8,S100A9,SERPINA3
GO:0016192	vesicle-mediated transport	2117	15	4.21	4.70E-06	0.00047	C4A,C4BPB,CRP,CSTB,EEF2,GNAI2,HBA2,HBB,ORM1,ORM2,S100A8,S100A9,SAA1,SERPINA3,UBB

GO:0036230	granulocyte activation	504	8	1	5.10E-06	0.00051	CSTB,EEF2,HBB,ORM1,ORM2,S100A8,S100A9,SERPINA3
GO:0006959	humoral immune response	361	7	0.72	5.90E-06	0.00059	APCS,C4A,C4BPB,CRP,H2BC12,S100A8,S100A9
GO:0031347	regulation of defense response	907	10	1.81	7.20E-06	0.00072	APCS,C4A,C4BPB,DDT,PSMB2,PSMB3,S100A8,S100A9,SAA1,UBB
GO:0043299	leukocyte degranulation	531	8	1.06	7.50E-06	0.00075	CSTB,EEF2,HBB,ORM1,ORM2,S100A8,S100A9,SERPINA3
GO:0002253	activation of immune response	717	9	1.43	8.10E-06	0.00081	APCS,C4A,C4BPB,CRP,PSMB2,PSMB3,S100A8,S100A9,UBB
GO:0002275	myeloid cell activation involved in immune response	541	8	1.08	8.60E-06	0.00086	CSTB,EEF2,HBB,ORM1,ORM2,S100A8,S100A9,SERPINA3
GO:0002444	myeloid leukocyte mediated immunity	547	8	1.09	9.30E-06	0.00093	CSTB,EEF2,HBB,ORM1,ORM2,S100A8,S100A9,SERPINA3
GO:0050896	response to stimulus	9377	31	18.66	9.70E-06	0.00097	ACTA2,APCS,ATP6V1G2,C4A,C4BPB,CA1,CRP,CSTB,DDT,EEF2,GNAI2,H2BC12,H4-16,H4C1,H6PD,HBA2,HBB,MYH7,ORM1,ORM2,OSBPL7,PSMB2,PSMB3,S100A8,S100A9,SAA1,SAA2,SERPINA3,UBB,VASP,YWHAG
GO:0045055	regulated exocytosis	792	9	1.58	1.80E-05	0.0018	CSTB,EEF2,GNAI2,HBB,ORM1,ORM2,S100A8,S100A9,SERPINA3
GO:0002274	myeloid leukocyte activation	656	8	1.31	3.50E-05	0.0035	CSTB,EEF2,HBB,ORM1,ORM2,S100A8,S100A9,SERPINA3
GO:0050778	positive regulation of immune response	888	9	1.77	4.40E-05	0.0044	APCS,C4A,C4BPB,CRP,PSMB2,PSMB3,S100A8,S100A9,UBB
GO:0050727	regulation of inflammatory response	502	7	1	5.00E-05	0.005	APCS,C4A,C4BPB,DDT,S100A8,S100A9,SAA1
GO:0006887	exocytosis	903	9	1.8	5.00E-05	0.005	CSTB,EEF2,GNAI2,HBB,ORM1,ORM2,S100A8,S100A9,SERPINA3
GO:0006810	transport	5239	22	10.43	5.10E-05	0.0051	ATP6V1G2,C4A,C4BPB,CA1,CRP,CSTB,EEF2,GNAI2,HBA2,HBB,ORM1,ORM2,OSBPL7,PSMB2,PSMB3,RPS28,S100A8,S100A9,SAA1,SERPINA3,UBB,YWHAG
GO:0002366	leukocyte activation involved in immune response	704	8	1.4	5.70E-05	0.0057	CSTB,EEF2,HBB,ORM1,ORM2,S100A8,S100A9,SERPINA3
GO:0002263	cell activation involved in immune response	708	8	1.41	5.90E-05	0.0059	CSTB,EEF2,HBB,ORM1,ORM2,S100A8,S100A9,SERPINA3
GO:0050716	positive regulation of interleukin-1 secretion	39	3	0.08	6.30E-05	0.0063	ORM1,ORM2,SAA1

GO:0051234	establishment of localization	5362	22	10.67	7.60E-05	0.0076	ATP6V1G2,C4A,C4BPB,CA1,CRP,CSTB,EEF2,GNAI2,HBA2,HBB,ORM1,ORM2,OSBPL7,PSMB2,PSMB3,RPS28,S100A8,S100A9,SAA1,SERPINA3,UBB,YWHAG
GO:0015701	bicarbonate transport	42	3	0.08	7.90E-05	0.0079	CA1,HBA2,HBB
GO:0065008	regulation of biological quality	4129	19	8.22	8.00E-05	0.008	ACTA2,ATP6V1G2,C4BPB,CRP,GNAI2,H4-16,H4C1,H6PD,HBB,MYH7,PSMB2,PSMB3,S100A8,S100A9,SAA1,SERPINA3,UBB,VASP,YWHAG
GO:0031323	regulation of cellular metabolic process	6329	24	12.6	8.90E-05	0.0089	ACTA2,APCS,ATP6V1G2,C4A,C4BPB,CRP,CSTB,DDT,EEF2,GNAI2,H2AC4,H4-16,H4C1,H6PD,HBB,OSBPL7,PSMB2,PSMB3,S100A8,S100A9,SAA1,SERPINA3,UBB,YWHAG
GO:0032119	sequestering of zinc ion	8	2	0.02	0.00011	0.011	S100A8,S100A9
GO:0019222	regulation of metabolic process	6988	25	13.91	0.00014	0.014	ACTA2,APCS,ATP6V1G2,C4A,C4BPB,CRP,CSTB,DDT,EEF2,GNAI2,H2AC4,H4-16,H4C1,H6PD,HBB,OSBPL7,PSMB2,PSMB3,RPS28,S100A8,S100A9,SAA1,SERPINA3,UBB,YWHAG
GO:0032940	secretion by cell	1563	11	3.11	0.00015	0.015	CRP,CSTB,EEF2,GNAI2,HBB,ORM1,ORM2,S100A8,S100A9,SAA1,SERPINA3
GO:0006958	complement activation, classical pathway	140	4	0.28	0.00017	0.017	APCS,C4A,C4BPB,CRP
GO:0030162	regulation of proteolysis	839	8	1.67	0.00019	0.019	C4A,C4BPB,CSTB,OSBPL7,S100A8,S100A9,SERPINA3,UBB
GO:0050704	regulation of interleukin-1 secretion	59	3	0.12	0.00022	0.022	ORM1,ORM2,SAA1
GO:0002455	humoral immune response mediated by circulating immunoglobulin	154	4	0.31	0.00024	0.024	APCS,C4A,C4BPB,CRP
GO:0032732	positive regulation of interleukin-1 production	61	3	0.12	0.00024	0.024	ORM1,ORM2,SAA1
GO:0009605	response to external stimulus	2595	14	5.16	0.00024	0.024	ACTA2,APCS,C4A,C4BPB,CRP,DDT,EEF2,GNAI2,H2BC12,S100A8,S100A9,SAA1,SAA2,VASP
GO:2001244	positive regulation of intrinsic apoptotic signaling pathway	62	3	0.12	0.00025	0.025	S100A8,S100A9,UBB
GO:0050776	regulation of immune response	1116	9	2.22	0.00025	0.025	APCS,C4A,C4BPB,CRP,PSMB2,PSMB3,S100A8,S100A9,UBB

GO:0070887	cellular response to chemical stimulus	3327	16	6.62	0.00027	0.027	ATP6V1G2,CA1,DDT,EEF2,GNAI2,HBA2,HBB,OSBPL7,PSMB2,PSMB3,S100A8,S100A9,SAA1,SAA2,UBB,YWHAG
GO:0002758	innate immune response-activating signal transduction	294	5	0.59	0.00027	0.027	PSMB2,PSMB3,S100A8,S100A9,UBB
GO:0035821	modification of morphology or physiology of other organism	162	4	0.32	0.00029	0.029	APCS,CRP,H2BC12,S100A9
GO:0008228	opsonization	13	2	0.03	0.0003	0.03	C4BPB,CRP
GO:0044793	negative regulation by host of viral process	13	2	0.03	0.0003	0.03	APCS,CRP
GO:0070486	leukocyte aggregation	13	2	0.03	0.0003	0.03	S100A8,S100A9
GO:0050701	interleukin-1 secretion	66	3	0.13	0.0003	0.03	ORM1,ORM2,SAA1
GO:0060326	cell chemotaxis	303	5	0.6	0.00031	0.031	DDT,S100A8,S100A9,SAA1,SAA2
GO:0046903	secretion	1703	11	3.39	0.00032	0.032	CRP,CSTB,EEF2,GNAI2,HBB,ORM1,ORM2,S100A8,S100A9,SAA1,SERPINA3
GO:0051179	localization	6792	24	13.52	0.00032	0.032	ATP6V1G2,C4A,C4BPB,CA1,CRP,CSTB,DDT,EEF2,GNAI2,HBA2,HBB,ORM1,ORM2,OSBPL7,PSMB2,PSMB3,RPS28,S100A8,S100A9,SAA1,SAA2,SERPINA3,UBB,YWHAG
GO:0002218	activation of innate immune response	316	5	0.63	0.00038	0.038	PSMB2,PSMB3,S100A8,S100A9,UBB
GO:0006956	complement activation	175	4	0.35	0.00039	0.039	APCS,C4A,C4BPB,CRP
GO:0002523	leukocyte migration involved in inflammatory response	15	2	0.03	0.0004	0.04	S100A8,S100A9
GO:0015671	oxygen transport	15	2	0.03	0.0004	0.04	HBA2,HBB
GO:0017014	protein nitrosylation	15	2	0.03	0.0004	0.04	S100A8,S100A9
GO:0018119	peptidyl-cysteine S-nitrosylation	15	2	0.03	0.0004	0.04	S100A8,S100A9
GO:0051238	sequestering of metal ion	15	2	0.03	0.0004	0.04	S100A8,S100A9
GO:0044419	interspecies interaction between organisms	937	8	1.86	0.00041	0.041	APCS,CRP,H2BC12,PSMB2,PSMB3,RPS28,S100A9,UBB
GO:0002684	positive regulation of immune system process	1194	9	2.38	0.00042	0.042	APCS,C4A,C4BPB,CRP,PSMB2,PSMB3,S100A8,S100A9,UBB
GO:0031349	positive regulation of defense response	507	6	1.01	0.00044	0.044	DDT,PSMB2,PSMB3,S100A8,S100A9,UBB

GO:2001235	positive regulation of apoptotic signaling pathway	182	4	0.36	0.00045	0.045	S100A8,S100A9,UBB,YWHAG
GO:0061418	regulation of transcription from RNA polymerase II promoter in response to hypoxia	78	3	0.16	0.0005	0.05	PSMB2,PSMB3,UBB

Table S5. 24 differentially expressed proteins between severe and moderate COVID-19 cases.

Gene name	Protein Accession	Ratio（Severe/Mild）	Up Or Down	Adjusted P-value	Protein Description
DDT	P30046	3.584	UP	0.0024	D-dopachrome decarboxylase
CAPNS1	P04632	2.996	UP	0.0155	Calpain small subunit 1
IgH	A0A2U8J946	2.790	UP	0.0416	Ig heavy chain variable region (Fragment)
TMSB4X	P62328	2.709	UP	0.0473	Thymosin beta-4
COL6A3	P12111	2.645	UP	0.0140	Collagen alpha-3(VI) chain
UBB	P0CG47	2.443	UP	0.0235	Polyubiquitin-B
VASP	P50552	2.319	UP	0.0221	Vasodilator-stimulated phosphoprotein
SOD2	P04179	2.228	UP	0.0025	Superoxide dismutase [Mn], mitochondrial
CSTB	P04080	2.156	UP	0.0098	Cystatin-B
H4C1	P62805	2.045	UP	0.0473	Histone H4
MB	P02144	1.976	UP	0.0075	Myoglobin
GSTO1	P78417	1.967	UP	0.0235	Glutathione S-transferase omega-1
ACTB	P60709	1.953	UP	0.0282	Actin, cytoplasmic 1
F8	P00451	1.899	UP	0.0137	Coagulation factor VIII
RPS28	P62857	1.770	UP	0.0154	40S ribosomal protein S28
S100A8	P05109	1.733	UP	0.0235	Protein S100-A8
S6C4Q9	S6C4Q9	1.733	UP	0.0173	IgG L chain
OAF	Q86UD1	1.608	UP	0.0282	Out at first protein homolog
S100A9	P06702	1.536	UP	0.0282	Protein S100-A9
TNFRSF17	Q02223	1.530	UP	0.0401	Tumor necrosis factor receptor superfamily member 17
CA6	P23280	0.636	DOWN	0.0336	Carbonic anhydrase 6
IGFALS	P35858	0.604	DOWN	0.0282	Insulin-like growth factor-binding protein complex acid labile subunit
HSPB1	P04792	0.436	DOWN	0.0084	Heat shock protein beta-1
VCPIP1	Q96JH7	0.242	DOWN	0.0303	Deubiquitinating protein VCIP135

Table S6. Significantly enriched GO biological process terms for severe COVID-19 patient-specific proteins.

GO.ID	Term	Annotated	Significant	Expected	P value	Adjusted P	Significant genes
GO:0070488	neutrophil aggregation	2	2	0	1.10E-06	0.00011	S100A8,S100A9
GO:0042592	homeostatic process	1950	10	2.11	9.90E-06	0.00099	ACTB,GSTO1,H4-16,H4C1,MB,S100A8,S100A9,SOD2,TNFRSF17,UBB
GO:0060326	cell chemotaxis	303	5	0.33	1.40E-05	0.0014	DDT,S100A8,S100A9,SAA2,TMSB4X
GO:0051262	protein tetramerization	145	4	0.16	1.60E-05	0.0016	H4-16,H4C1,SOD2,VASP
GO:0009636	response to toxic substance	544	6	0.59	1.60E-05	0.0016	ACTB,GSTO1,MB,S100A8,S100A9,SOD2
GO:2001242	regulation of intrinsic apoptotic signaling pathway	168	4	0.18	2.80E-05	0.0028	S100A8,S100A9,SOD2,UBB
GO:0032119	sequestering of zinc ion	8	2	0.01	3.10E-05	0.0031	S100A8,S100A9
GO:2001244	positive regulation of intrinsic apoptotic signaling pathway	62	3	0.07	3.90E-05	0.0039	S100A8,S100A9,UBB
GO:0065008	regulation of biological quality	4129	13	4.46	4.10E-05	0.0041	ACTB,F8,GSTO1,H4-16,H4C1,MB,S100A8,S100A9,SOD2,TMSB4X,TNFRSF17,UBB,VASP
GO:0006935	chemotaxis	647	6	0.7	4.40E-05	0.0044	DDT,S100A8,S100A9,SAA2,TMSB4X,VASP
GO:0042330	taxis	649	6	0.7	4.40E-05	0.0044	DDT,S100A8,S100A9,SAA2,TMSB4X,VASP
GO:0032501	multicellular organismal process	7828	17	8.46	6.00E-05	0.006	ACTB,CAPNS1,COL6A3,CSTB,DDT,F8,GSTO1,H4-16,H4C1,MB,S100A8,S100A9,SOD2,TMSB4X,TNFRSF17,UBB,VASP
GO:0070486	leukocyte aggregation	13	2	0.01	8.60E-05	0.0086	S100A8,S100A9
GO:0002523	leukocyte migration involved in inflammatory response	15	2	0.02	0.00012	0.012	S100A8,S100A9
GO:0017014	protein nitrosylation	15	2	0.02	0.00012	0.012	S100A8,S100A9
GO:0018119	peptidyl-cysteine S-nitrosylation	15	2	0.02	0.00012	0.012	S100A8,S100A9
GO:0051238	sequestering of metal ion	15	2	0.02	0.00012	0.012	S100A8,S100A9
GO:0043044	ATP-dependent chromatin remodeling	90	3	0.1	0.00012	0.012	ACTB,H4-16,H4C1
GO:0097237	cellular response to toxic substance	254	4	0.27	0.00014	0.014	ACTB,GSTO1,S100A9,SOD2
GO:0045653	negative regulation of megakaryocyte differentiation	18	2	0.02	0.00017	0.017	H4-16,H4C1
GO:0098869	cellular oxidant detoxification	103	3	0.11	0.00018	0.018	GSTO1,S100A9,SOD2
GO:0006954	inflammatory response	875	6	0.95	0.00023	0.023	DDT,F8,S100A8,S100A9,SAA2,TMSB4X
GO:0097193	intrinsic apoptotic signaling pathway	291	4	0.31	0.00023	0.023	S100A8,S100A9,SOD2,UBB
GO:1990748	cellular detoxification	114	3	0.12	0.00024	0.024	GSTO1,S100A9,SOD2
GO:0006928	movement of cell or subcellular component	2239	9	2.42	0.00024	0.024	ACTB,DDT,S100A8,S100A9,SAA2,SOD2,TMSB4X,UBB,VASP

GO:0006950	response to stress	4178	12	4.51	0.00029	0.029	ACTB,DDT,F8,H4-16,H4C1,MB,S100A8,S100A9,SAA2,SOD2,TMSB4X,UBB
GO:0065003	protein-containing complex assembly	1824	8	1.97	0.00035	0.035	F8,H4-16,H4C1,RPS28,SOD2,TMSB4X,UBB,VASP
GO:0032103	positive regulation of response to external stimulus	325	4	0.35	0.00036	0.036	DDT,S100A8,S100A9,TMSB4X
GO:0098754	detoxification	132	3	0.14	0.00037	0.037	GSTO1,S100A9,SOD2
GO:0051235	maintenance of location	347	4	0.37	0.00046	0.046	GSTO1,S100A8,S100A9,TMSB4X

Table S7. Average AUCs for random forest model built with different combinations of proteins.

Rank	Combinations							AUC
1	DDT	OAF	MB					0.907828306
2	DDT	OAF						0.907392305
3	DDT	MB						0.907187658
4	DDT							0.898882144
5	DDT	OAF	MB	CAPNS1				0.866322447
6	DDT	OAF	GST01					0.866028503
7	DDT	OAF	MB	S100A8				0.860263131
8	DDT	MB	CAPNS1					0.859293179
9	DDT	MB	GST01					0.857223134
10	DDT	GST01						0.855488793
11	DDT	OAF	RPS28					0.850739227
12	DDT	MB	SOD2					0.850694994
13	DDT	MB	RPS28					0.850470514
14	DDT	OAF	CAPNS1					0.847262665
15	DDT	OAF	MB	RPS28				0.845455766
16	DDT	SOD2						0.839511921
17	DDT	MB	S100A8					0.83535057
18	DDT	OAF	SOD2					0.834464108
19	DDT	OAF	MB	GST01				0.827705407
20	DDT	OAF	MB	RPS28	CAPNS1			0.818725293
21	DDT	MB	GST01	CAPNS1				0.814385897
22	OAF							0.81427454
23	DDT	GST01	CAPNS1					0.813774859
24	DDT	MB	RPS28	CAPNS1				0.813433477
25	DDT	OAF	MB	S100A8	CAPNS1			0.812423929
26	DDT	OAF	MB	GST01	RPS28			0.811800361
27	DDT	MB	GST01	RPS28				0.810852845
28	DDT	GST01	SOD2					0.810711752
29	DDT	OAF	MB	GST01	CAPNS1			0.809104178
30	DDT	RPS28						0.809059756
31	DDT	GST01	RPS28					0.80891727
32	DDT	OAF	S100A8					0.804156987
33	DDT	OAF	GST01	CAPNS1				0.8039121
34	OAF	MB						0.802585903
35	DDT	OAF	MB	GST01	S100A8			0.801273896
36	DDT	MB	GST01	S100A8				0.801249975
37	DDT	OAF	MB	RPS28	S100A8			0.801029009
38	DDT	MB	S100A8	CAPNS1				0.80071682
39	DDT	CAPNS1						0.800632488
40	DDT	OAF	GST01	RPS28				0.800554238
41	DDT	SOD2	CAPNS1					0.79805591

42	DDT	OAF	RPS28	CAPNS1					0.798017799
43	DDT	MB	SOD2	CAPNS1					0.797005413
44	DDT	MB	GST01	RPS28	CAPNS1				0.793985771
45	DDT	OAF	MB	GST01	RPS28	CAPNS1			0.793182509
46	DDT	OAF	MB	SOD2					0.78920716
47	DDT	MB	RPS28	S100A8					0.788673194
48	DDT	MB	GST01	SOD2	CAPNS1				0.788188287
49	DDT	OAF	MB	SOD2	CAPNS1				0.78658846
50	DDT	MB	S100A8	SOD2					0.785652593
51	DDT	OAF	MB	GST01	RPS28	S100A8			0.785066391
52	DDT	OAF	MB	GST01	S100A8	CAPNS1			0.783144601
53	DDT	OAF	GST01	RPS28	CAPNS1				0.782316285
54	DDT	MB	S100A8	SOD2	CAPNS1				0.781208386
55	DDT	MB	GST01	RPS28	SOD2	CAPNS1			0.779990675
56	DDT	OAF	GST01	S100A8					0.779888098
57	DDT	OAF	MB	RPS28	S100A8	CAPNS1			0.779545906
58	DDT	MB	GST01	S100A8	CAPNS1				0.778763405
59	DDT	MB	GST01	SOD2					0.777923523
60	DDT	MB	RPS28	SOD2	CAPNS1				0.77719597
61	DDT	GST01	S100A8						0.7764239
62	DDT	OAF	MB	GST01	SOD2				0.77600073
63	DDT	OAF	MB	GST01	SOD2	CAPNS1			0.775883797
64	DDT	MB	GST01	RPS28	SOD2				0.77496733
65	DDT	MB	GST01	RPS28	S100A8				0.774755867
66	DDT	OAF	MB	S100A8	SOD2				0.77467737
67	DDT	OAF	S100A8	CAPNS1					0.774385323
68	DDT	OAF	MB	GST01	RPS28	SOD2	CAPNS1		0.7728787
69	DDT	OAF	MB	GST01	RPS28	SOD2			0.77218107
70	DDT	OAF	SOD2	CAPNS1					0.77168035
71	DDT	MB	GST01	S100A8	SOD2	CAPNS1			0.771374648
72	DDT	RPS28	SOD2						0.771275077
73	DDT	OAF	MB	GST01	S100A8	SOD2	CAPNS1		0.771216931
74	DDT	MB	GST01	S100A8	SOD2				0.770652557
75	DDT	OAF	MB	GST01	RPS28	S100A8	CAPNS1		0.770219141
76	OAF	MB	CAPNS1						0.770145595
77	DDT	OAF	MB	S100A8	SOD2	CAPNS1			0.768799286
78	DDT	OAF	GST01	SOD2	CAPNS1				0.768390196
79	DDT	OAF	MB	RPS28	SOD2				0.768064725
80	DDT	OAF	GST01	RPS28	SOD2	CAPNS1			0.767692241
81	DDT	OAF	MB	GST01	S100A8	SOD2			0.767256989
82	DDT	GST01	SOD2	CAPNS1					0.766782623
83	DDT	MB	RPS28	SOD2					0.76596032
84	DDT	MB	RPS28	S100A8	CAPNS1				0.76537624
85	DDT	OAF	GST01	S100A8	CAPNS1				0.765200862

86	OAF	MB	GST01						0.765165319
87	DDT	MB	GST01	RPS28	S100A8	SOD2	CAPNS1		0.764282673
88	DDT	OAF	MB	RPS28	SOD2	CAPNS1			0.763594523
89	DDT	GST01	RPS28	SOD2	CAPNS1				0.762486769
90	OAF	MB	RPS28						0.76155365
91	MB	SOD2	CAPNS1						0.761505403
92	DDT	MB	RPS28	S100A8	SOD2	CAPNS1			0.761381743
93	DDT	GST01	RPS28	CAPNS1					0.761367147
94	DDT	OAF	MB	GST01	RPS28	S100A8	SOD2		0.759840137
95	DDT	OAF	GST01	SOD2					0.759795658
96	DDT	OAF	MB	GST01	RPS28	S100A8	SOD2	CAPNS1	0.759636725
97	DDT	OAF	GST01	RPS28	S100A8				0.759603073
98	OAF	GST01							0.758995502
99	DDT	OAF	RPS28	S100A8					0.758562101
100	DDT	MB	GST01	RPS28	S100A8	SOD2			0.758559773
101	GST01	SOD2	CAPNS1						0.758495814
102	MB	GST01	CAPNS1						0.758293499
103	DDT	OAF	MB	RPS28	S100A8	SOD2			0.757665422
104	DDT	OAF	GST01	RPS28	SOD2				0.757631278
105	DDT	GST01	RPS28	SOD2					0.756429686
106	DDT	MB	GST01	RPS28	S100A8	CAPNS1			0.755988465
107	DDT	OAF	MB	RPS28	S100A8	SOD2	CAPNS1		0.755074714
108	DDT	OAF	GST01	S100A8	SOD2	CAPNS1			0.754574388
109	MB	GST01	SOD2						0.754538492
110	DDT	MB	RPS28	S100A8	SOD2				0.753637157
111	DDT	S100A8	SOD2						0.753364958
112	MB	RPS28	CAPNS1						0.752189511
113	DDT	GST01	RPS28	S100A8					0.75162962
114	DDT	OAF	GST01	S100A8	SOD2				0.751552636
115	DDT	S100A8	SOD2	CAPNS1					0.75094518
116	DDT	RPS28	SOD2	CAPNS1					0.750049667
117	DDT	OAF	GST01	RPS28	S100A8	CAPNS1			0.749478134
118	DDT	RPS28	CAPNS1						0.749255807
119	DDT	GST01	S100A8	CAPNS1					0.748941135
120	MB	CAPNS1							0.748651683
121	DDT	GST01	S100A8	SOD2	CAPNS1				0.748463113
122	OAF	MB	SOD2						0.747652571
123	DDT	OAF	RPS28	SOD2	CAPNS1				0.747425259
124	DDT	OAF	GST01	RPS28	S100A8	SOD2			0.747315783
125	MB	GST01	SOD2	CAPNS1					0.747281721
126	DDT	OAF	GST01	RPS28	S100A8	SOD2	CAPNS1		0.746879118
127	DDT	GST01	RPS28	S100A8	SOD2	CAPNS1			0.746689155
128	OAF	GST01	CAPNS1						0.746449756
129	DDT	GST01	S100A8	SOD2					0.746043516

130	MB	GST01	RPS28	SOD2	CAPNS1				0.745296681
131	OAF	GST01	SOD2						0.745122392
132	GST01	SOD2							0.743928723
133	OAF	GST01	RPS28						0.742934045
134	DDT	OAF	S100A8	SOD2	CAPNS1				0.742768755
135	OAF	MB	RPS28	CAPNS1					0.742620926
136	OAF	MB	S100A8						0.742014086
137	DDT	OAF	S100A8	SOD2					0.741406475
138	DDT	GST01	RPS28	S100A8	SOD2				0.741340898
139	OAF	MB	GST01	RPS28	CAPNS1				0.740989682
140	GST01	RPS28	SOD2	CAPNS1					0.739350821
141	MB	GST01	S100A8	SOD2	CAPNS1				0.738499927
142	MB	S100A8	SOD2	CAPNS1					0.738324161
143	MB	GST01							0.738161587
144	MB	S100A8	SOD2						0.737049953
145	OAF	MB	GST01	S100A8	SOD2	CAPNS1			0.736947435
146	OAF	MB	GST01	RPS28					0.736216019
147	OAF	MB	GST01	CAPNS1					0.735943563
148	DDT	GST01	RPS28	S100A8	CAPNS1				0.735549474
149	MB	SOD2							0.735418516
150	OAF	MB	GST01	RPS28	SOD2	CAPNS1			0.735307831
151	DDT	OAF	RPS28	SOD2					0.734770622
152	OAF	MB	GST01	SOD2	CAPNS1				0.733116361
153	DDT	OAF	RPS28	S100A8	CAPNS1				0.732411356
154	MB	GST01	RPS28						0.732394672
155	GST01	RPS28	SOD2						0.73059433
156	OAF	MB	GST01	RPS28	S100A8	SOD2	CAPNS1		0.730503149
157	DDT	S100A8							0.730130349
158	MB	GST01	RPS28	S100A8	SOD2	CAPNS1			0.729909588
159	MB	GST01	S100A8	SOD2					0.729572855
160	OAF	GST01	RPS28	SOD2	CAPNS1				0.72950707
161	MB	RPS28	S100A8	SOD2	CAPNS1				0.727526202
162	MB	RPS28	SOD2						0.727370107
163	MB	RPS28	SOD2	CAPNS1					0.727366458
164	OAF	GST01	SOD2	CAPNS1					0.727201617
165	SOD2	CAPNS1							0.726776136
166	MB	GST01	RPS28	SOD2					0.726644054
167	GST01	S100A8	SOD2	CAPNS1					0.726566453
168	OAF	MB	GST01	RPS28	SOD2				0.72610109
169	OAF	GST01	RPS28	CAPNS1					0.725760567
170	MB	GST01	RPS28	S100A8	SOD2				0.724780467
171	MB	GST01	RPS28	CAPNS1					0.724205056
172	OAF	MB	S100A8	CAPNS1					0.723582071
173	DDT	OAF	RPS28	S100A8	SOD2	CAPNS1			0.723322184

174	DDT	RPS28	S100A8	SOD2	CAPNS1				0.722744025
175	OAF	GST01	S100A8	SOD2	CAPNS1				0.722024772
176	OAF	MB	GST01	RPS28	S100A8	CAPNS1			0.721227119
177	OAF	MB	GST01	RPS28	S100A8	SOD2			0.721173752
178	GST01	RPS28	S100A8	SOD2	CAPNS1				0.720651138
179	MB	RPS28							0.720553427
180	RPS28	SOD2	CAPNS1						0.720175238
181	OAF	MB	GST01	S100A8	CAPNS1				0.72017485
182	OAF	MB	GST01	S100A8	SOD2				0.720055345
183	OAF	MB	GST01	S100A8					0.719627805
184	OAF	MB	GST01	RPS28	S100A8				0.718771919
185	OAF	GST01	RPS28	S100A8	SOD2	CAPNS1			0.71836364
186	MB	GST01	S100A8						0.718116321
187	OAF	MB	GST01	SOD2					0.717488293
188	OAF	MB	SOD2	CAPNS1					0.717304779
189	GST01	S100A8	SOD2						0.716795394
190	MB	GST01	S100A8	CAPNS1					0.716245914
191	OAF	GST01	RPS28	SOD2					0.716150743
192	OAF	MB	RPS28	S100A8	SOD2	CAPNS1			0.715721332
193	OAF	MB	RPS28	SOD2	CAPNS1				0.715278234
194	OAF	MB	RPS28	S100A8					0.713946598
195	OAF	CAPNS1							0.713669951
196	OAF	MB	S100A8	SOD2	CAPNS1				0.713218999
197	S100A8	SOD2	CAPNS1						0.712189325
198	DDT	OAF	RPS28	S100A8	SOD2				0.710813923
199	MB								0.709831808
200	OAF	GST01	S100A8	SOD2					0.709577328
201	GST01	RPS28	S100A8	SOD2					0.709383121
202	MB	GST01	RPS28	S100A8	CAPNS1				0.70832492
203	GST01	CAPNS1							0.708240183
204	OAF	MB	RPS28	S100A8	CAPNS1				0.707955067
205	OAF	SOD2							0.7077273
206	OAF	RPS28							0.706656125
207	OAF	GST01	S100A8						0.70633461
208	OAF	GST01	RPS28	S100A8	SOD2				0.706307851
209	OAF	SOD2	CAPNS1						0.706158649
210	OAF	RPS28	CAPNS1						0.703650996
211	MB	RPS28	S100A8	SOD2					0.70149475
212	DDT	RPS28	S100A8	SOD2					0.701237406
213	OAF	MB	S100A8	SOD2					0.700669463
214	MB	GST01	RPS28	S100A8					0.699990067
215	OAF	GST01	S100A8	CAPNS1					0.699853489
216	OAF	GST01	RPS28	S100A8	CAPNS1				0.698259442
217	MB	S100A8	CAPNS1						0.69584734

218	OAF	GST01	RPS28	S100A8					0.695156196
219	MB	RPS28	S100A8	CAPNS1					0.693648361
220	RPS28	S100A8	SOD2	CAPNS1					0.692696791
221	OAF	RPS28	SOD2	CAPNS1					0.691199092
222	DDT	S100A8	CAPNS1						0.690732274
223	OAF	MB	RPS28	S100A8	SOD2				0.690238322
224	OAF	RPS28	SOD2						0.690130755
225	MB	S100A8							0.687923939
226	OAF	MB	RPS28	SOD2					0.68632042
227	GST01	RPS28	CAPNS1						0.685641141
228	S100A8	SOD2							0.684085364
229	RPS28	SOD2							0.683179063
230	OAF	S100A8	SOD2	CAPNS1					0.683168897
231	MB	RPS28	S100A8						0.673454096
232	DDT	RPS28	S100A8	CAPNS1					0.672750715
233	OAF	RPS28	S100A8	SOD2	CAPNS1				0.671234771
234	GST01	RPS28	S100A8	CAPNS1					0.670242251
235	OAF	S100A8	SOD2						0.669742342
236	GST01	RPS28							0.66859089
237	GST01	S100A8	CAPNS1						0.663928723
238	DDT	RPS28	S100A8						0.663534692
239	GST01	RPS28	S100A8						0.65875525
240	GST01	S100A8							0.658302824
241	OAF	RPS28	S100A8	SOD2					0.651892801
242	SOD2								0.650667291
243	RPS28	S100A8	SOD2						0.650095372
244	OAF	S100A8							0.649221612
245	GST01								0.648652382
246	OAF	RPS28	S100A8	CAPNS1					0.643721138
247	OAF	S100A8	CAPNS1						0.639571907
248	RPS28	CAPNS1							0.621672444
249	OAF	RPS28	S100A8						0.618244035
250	CAPNS1								0.598815887
251	RPS28								0.547484022
252	RPS28	S100A8	CAPNS1						0.506007866
253	S100A8	CAPNS1							0.473938657
254	RPS28	S100A8							0.448705771
255	S100A8								0.359768088

Data S1. (separate file)

List of LC-MS/MS detected peptides and proteins from sera of 23 COVID-19 patients (Development group) and 10 healthy controls
